# Neural stem cell–specific ITPA deficiency causes neural depolarization and epilepsy

**DOI:** 10.1172/jci.insight.140229

**Published:** 2020-11-19

**Authors:** Yuichiro Koga, Daisuke Tsuchimoto, Yoshinori Hayashi, Nona Abolhassani, Yasuto Yoneshima, Kunihiko Sakumi, Hiroshi Nakanishi, Shinya Toyokuni, Yusaku Nakabeppu

**Affiliations:** 1 Division of Neurofunctional Genomics, Department of Immunobiology and Neuroscience, Medical Institute of Bioregulation, and; 2 Department of Aging Science and Pharmacology, Faculty of Dental Sciences, Kyushu University, Fukuoka, Japan.; 3 Department of Physiology, Nihon University School of Dentistry, Tokyo, Japan.; 4 Department of Pharmacology, Faculty of Pharmacy, Yasuda Women’s University, Hiroshima, Japan.; 5 Department of Pathology and Biological Responses, Nagoya University Graduate School of Medicine, Nagoya, Japan.

**Keywords:** Neuroscience, Epilepsy

## Abstract

Inosine triphosphate pyrophosphatase (ITPA) hydrolyzes inosine triphosphate (ITP) and other deaminated purine nucleotides to the corresponding nucleoside monophosphates. In humans, ITPA deficiency causes severe encephalopathy with epileptic seizure, microcephaly, and developmental retardation. In this study, we established neural stem cell–specific *Itpa*–conditional KO mice (*Itpa*-cKO mice) to clarify the effects of ITPA deficiency on the neural system. The *Itpa*-cKO mice showed growth retardation and died within 3 weeks of birth. We did not observe any microcephaly in the *Itpa*-cKO mice, although the female *Itpa*-cKO mice did show adrenal hypoplasia. The *Itpa*-cKO mice showed limb-clasping upon tail suspension and spontaneous and/or audiogenic seizure. Whole-cell patch-clamp recordings from entorhinal cortex neurons in brain slices revealed a depolarized resting membrane potential, increased firing, and frequent spontaneous miniature excitatory postsynaptic current and miniature inhibitory postsynaptic current in the *Itpa*-cKO mice compared with ITPA-proficient controls. Accumulated ITP or its metabolites, such as cyclic inosine monophosphates, or RNA containing inosines may cause membrane depolarization and hyperexcitability in neurons and induce the phenotype of ITPA-deficient mice, including seizure.

## Abstract 2

Neural stem cell-specific knockout of inosine triphosphate pyrophosphatase causes depolarization of neuronal resting membrane potential, epileptic seizure, postnatal growth retardation, and early death in mice.

## Graphical abstract

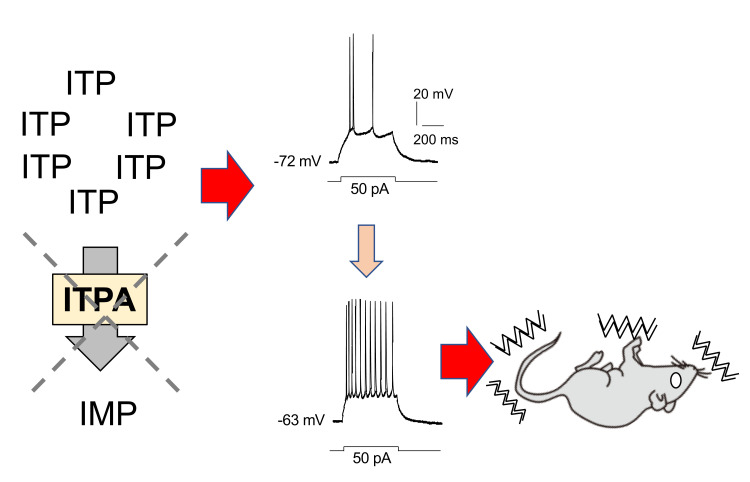

## Introduction

The base moieties of nucleotides are damaged by reactive molecules, such as reactive oxygen and nitrogen species, which are generated via the normal metabolism or through exposure to ionizing radiation and chemicals under physiological conditions. Noncanonical nucleotides with damaged base moieties are presumed to compete with normal nucleotides and cause deleterious effects on organisms (1–4).

The incorporation of noncanonical deoxynucleoside triphosphates into newly synthesized DNA during DNA replication may induce cell death because of the instability of DNA or genetic mutation followed by aging or oncogenesis (2, 4–6). For example, the incorporation of 8-oxo-2′-deoxyguanosine triphosphate, which is formed by the spontaneous oxidation of deoxyguanosine triphosphate (dGTP) into DNA, can cause a transversion mutation, as 8-oxoguanine can oppose both adenine and cytosine (7–10). The accumulation of noncanonical ribonucleoside triphosphates in intracellular nucleotide pools may cause competition with adenosine triphosphate (ATP) or guanosine triphosphate (GTP) on ATP- or GTP-dependent enzymes or induce changes in the RNA function via their incorporation into RNA.

Therefore, to avoid unfavorable effects of the noncanonical nucleotides, cells are equipped with specific enzymes to hydrolyze the noncanonical nucleoside triphosphates to their corresponding monophosphates and pyrophosphates.

Inosine triphosphate (ITP) and deoxyinosine triphosphate (dITP) are noncanonical nucleotides generated by the oxidative deamination of the adenine bases of ATP and deoxyadenosine triphosphate (dATP), respectively. Two-step phosphorylation of cellular inosine monophosphate, a physiological intermediate molecule of purine nucleotide biosynthesis, also generates ITP (11). Mammalian cells are equipped with inosine triphosphate pyrophosphatase (ITPA), which is encoded by the *ITPA* gene, to eliminate ITP, dITP, xanthosine triphosphate, and deoxyxanthosine triphosphate. ITPA helps sanitize nucleotide pools by hydrolyzing these nucleoside triphosphates to the corresponding purine nucleoside monophosphates and pyrophosphates (12, 13).

The single-nucleotide polymorphism (SNP) of human *ITPA* (rs1127354, 94C>A), which is a Pro32 to Thr (P32T) missense mutation, leads to reduced enzymatic ITPA activity via protein instability, a decreased rate of catalysis, and abnormal mRNA splicing (14–16). Homozygosity for the 94C>A polymorphism on human *ITPA* causes the abnormal accumulation of ITP in erythrocytes and is associated with increased drug toxicity of purine analogs (17, 18) and decreased drug toxicity of ribavirin, which is an antiviral drug (19). In humans, it was recently reported that patients with homozygous loss-of-function mutations in the *ITPA* gene showed severe encephalopathy with epileptic seizure and microcephaly or dilated cardiomyopathy. Furthermore, all patients showed developmental retardation, and most died before 4 years of age (3, 20–23).

At present, encephalopathy due to ITPA deficiency is described as “early infantile epileptic encephalopathy 35 (EIEE35)” in the Online Mendelian Inheritance in Man (OMIM) database (OMIM #616647). We previously established *Itpa*-knockout (KO) mice and reported that some died before birth, and the rest died about 2 weeks after birth with features of growth retardation (24). A significant amount of ITP was observed in the nucleotide pool of the *Itpa*-KO mouse erythrocytes but not in that of WT mouse erythrocytes (24). Primary mouse embryonic fibroblasts (pMEFs) derived from *Itpa*-KO mice were deficient in ITPA activity, and the inosine levels in cellular RNA and deoxyinosine levels in nuclear DNA of KO pMEFs were increased compared with WT pMEFs (25). While we deduced that the accumulation of ITP, the oxidatively deaminated product of ATP, caused deleterious effects on neurons consuming large amounts of ATP, we were unable to thoroughly analyze the influence of ITPA deficiency on the neural system because the *Itpa*-KO mice died in the early postnatal period due to severe heart failure.

In the present study, we generated neural stem cell–specific *Itpa*-KO mice (*Itpa^fl/fl^/Nes-Cre*) in order to analyze the influence of ITPA deficiency on the central neural system. *Itpa* KO in the neural stem cells is expected to result in ITPA deficiency in neurons and glial cells, except for microglia (26).

## Results

### Preparation of neural stem cell–specific Itpa–conditional KO mice.

As described in the Methods section, we established a mouse line carrying the *Itpa^fl^* allele and then prepared *Itpa^fl/fl^/Nes-Cre* mice in which the *Itpa* gene was disrupted only in the neural stem cell lineage by mating *Itpa^fl/fl^* mice with *Itpa^+/fl^/Nes-Cre* mice. The resulting mouse line was designated neural stem cell–specific *Itpa*-conditional knockout (*Itpa*-cKO) (Supplemental Figure 1, A–D; supplemental material available online with this article; https://doi.org/10.1172/jci.insight.140229DS1).

**Figure 1:**
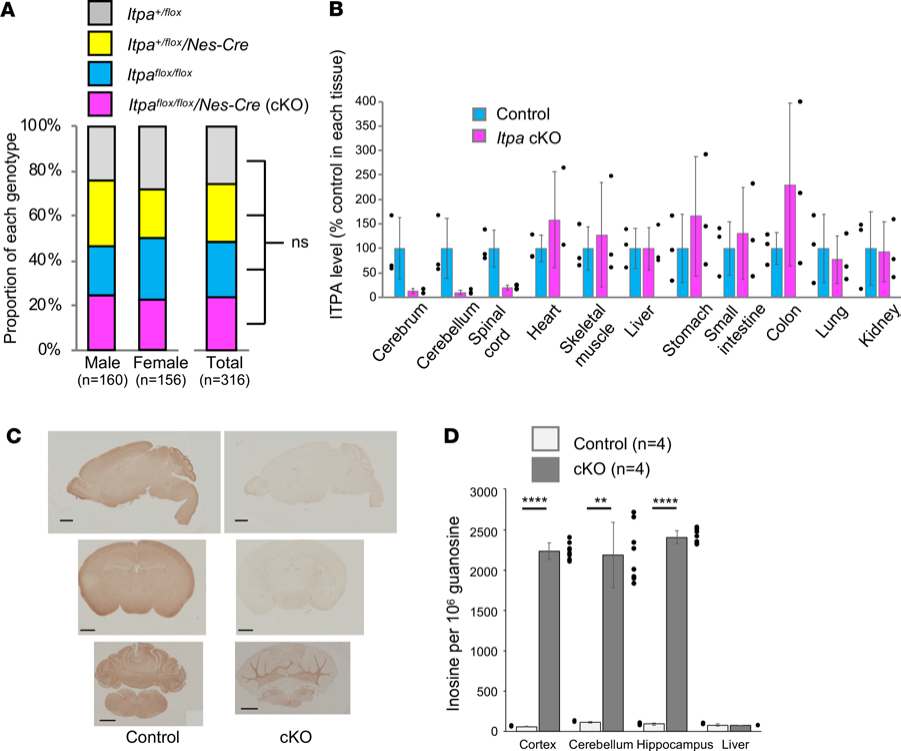
Generation and confirmation of neural stem cell–specific *Itpa*-cKO mice. (**A**) Birth ratio of the obtained mice. A total of 316 mice were obtained by the mating shown in Supplemental Figure 1D, and the ratios of the 4 genotypes in male, female, and total mice are shown. Statistical analyses were performed with Pearson’s χ^2^ test. Male: *P* = 0.55; female: *P* = 0.59; total: *P* = 0.90; ns: not significant (*P* > 0.05). (**B**) ITPA protein expression in P16 or P17 male *Itpa*-cKO (*Itpa^fl/fl^/Nes-Cre*) and control (*Itpa^fl/fl^*) mouse tissues. The ITPA protein levels in the tissue extracts from 3 *Itpa*-cKO and 3 control male mice were detected by Western blotting with anti-ITPA antiserum, quantified using cerebrum extract from 1 control mouse as a common standard to make a standard curve, and normalized by GAPDH levels. The ITPA expression levels in *Itpa*-cKO relative to those in control samples are shown for each tissue as the mean ± SD. Five micrograms of total protein of each sample was loaded in each lane. The original blot images are shown in Supplemental Figure 2. Statistical analyses were performed with Welch’s *t* test. *P* > 0.05 for each tissue. (**C**) Immunohistochemistry of P16 male mouse brains with anti-ITPA antibody. Images of sagittal (upper), coronal including cerebrum (middle), and coronal including cerebellum (lower) sections of paraffin-embedded brains of control male mice (left upper, *Itpa^+/fl^*/ *Nes-Cre*; left middle, *Itpa^+/fl^*; left lower, *Itpa^+/fl^*/*Nes-Cre*) and *Itpa*-cKO male mice (right) are shown. Scale bar: 1 mm. (**D**) Inosine content in RNA from brain subregions and liver. The inosine content in the total RNA samples that did not contain any small RNA molecules extracted from the cerebral cortex, cerebellum, hippocampal formation, and liver of P16 control mice (*Itpa^fl/fl^*) and *Itpa*-cKO mice are shown as the average inosine content per 10^6^ guanosine with standard deviations (*n* = 4). Statistical analyses were performed with Welch’s *t* test. Cerebral cortex: ****P < 0.0001; cerebellum: **P = 0.0019; hippocampal formation: ****P < 0.0001; liver: *P* = 0.30.

By this mating, we expected to obtain mice with 4 genotypes, including *Itpa^fl/fl^/Nes-Cre*, as shown in Supplemental Figure 1D. Following Mendel’s law, mice with each genotype were born at almost equal proportions (around 25%), with 160 male and 156 female mice born, indicating no obvious embryonic lethality in *Itpa^fl/fl^/Nes-Cre* mice (Figure 1A).

### The expression of ITPA protein in Itpa-cKO mice.

The ITPA protein levels in various tissues of *Itpa^fl/fl^/Nes-Cre* (*Itpa*-cKO) and *Itpa^fl/fl^* (control) male mice were quantified by Western blotting using anti-ITPA rabbit antiserum (13) and normalized by GAPDH levels. Relative ITPA levels to the mean level in control in each tissue are shown in Figure 1B. The *Itpa*-cKO mice showed a reduction in the ITPA protein level in the cerebrum, cerebellum, and spinal cord, although the differences were not statistically significant (*P* = 0.136, *P* = 0.1248, *P* = 0.0640, respectively). In other tissues, no reduction of ITPA levels was observed, suggesting a neural system–specific ITPA deficiency in *Itpa*-cKO mice. Full uncut images of all blots are shown in Supplemental Figure 2. The cerebrum, cerebellum, and spinal cord of *Itpa*-cKO mice were found to retain the signals of ITPA protein. Those signals were likely derived from cells that did not originate from neural stem cells, such as microglia or vascular endothelial cells. Immunohistochemistry analyses of paraffin-embedded thin brain sections using partially purified anti-ITPA rabbit antibody also indicated a reduction in the ITPA protein signals in all areas, including the cerebrum, hippocampus, dentate gyrus, and cerebellum, of *Itpa*-cKO mice compared with control mice (Figure 1C for male and Supplemental Figure 3 for female).

### The accumulation of inosine in RNA of Itpa-cKO mice.

The accumulation of ITP in nucleotide pools of ITPA-deficient cells was expected to result in an increase in the inosine content in RNA via the incorporation of ITP during transcription. Therefore, we measured the inosine levels in total RNA from mouse tissues as described in the Methods section. In brief, we hydrolyzed and dephosphorylated RNA extracted from mouse tissues to nucleosides and measured their inosine content by high-performance liquid chromatography–tandem mass spectrometry (HPLC-MS/MS). The results showed that the ratio of inosine to guanosine in RNA from the cerebral cortex, hippocampus, and cerebellum of *Itpa*-cKO mice was significantly higher (about 20-fold) than that in *Itpa^fl/fl^* control mice (*P* < 0.0001) (Figure 1D). This finding indicated the accumulation of ITP in ITPA-deficient cells, resulting in the incorporation of ITP into RNA. In contrast, the inosine levels in RNA from *Itpa*-cKO mouse livers, which expressed ITPA normally, as shown in Figure 1B, were comparable to those in ITPA-proficient control animals. This result supported the neural system–specific loss of the ITPA function.

### The growth and survival.

**Figure 2:**
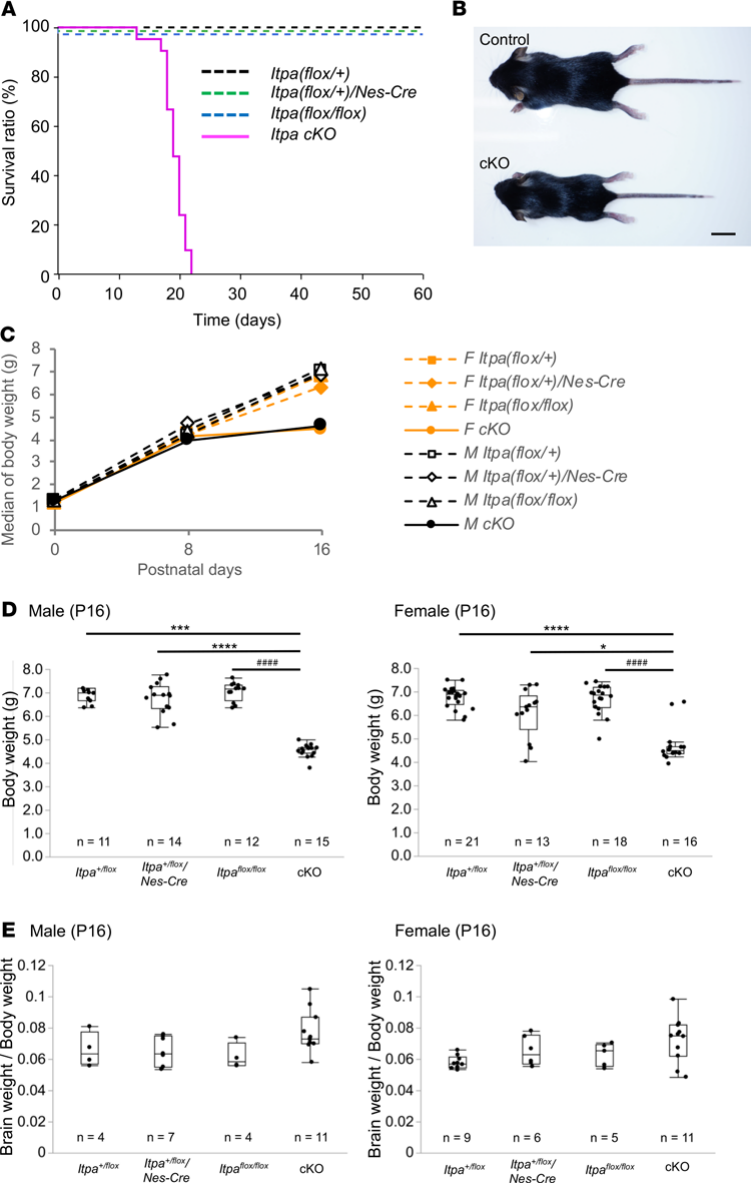
Growth retardation and early death of neural stem cell–specific *Itpa*-cKO mice. (**A**) *Itpa*-cKO mice died about 3 weeks after birth. The survival curves during 60 days after birth are shown as a Kaplan-Meier plot. *Itpa*-cKO mice, solid line; other ITPA-proficient control mice, broken lines. Statistical analyses were performed using the Log rank test with Bonferroni’s adjustment; *Itpa^fl/fl^/Nes-Cre* (*Itpa*-cKO, 10 female and 11 male) vs. *Itpa^+/fl^* (8 female and 6 male), *P* < 0.0001 (Bonferroni-adjusted *P* < 0.001); *Itpa*-cKO vs. *Itpa^+/fl^/Nes-Cre* (10 female and 12 male), *P* < 0.0001 (Bonferroni-adjusted *P* < 0.001); *Itpa*-cKO vs. *Itpa^fl/fl^* (12 female and 13 male), *P* < 0.0001 (Bonferroni-adjusted *P* < 0.001). (**B**) Small body size of *Itpa*-cKO mice. Images of *Itpa*-cKO (*Itpa^fl/fl^/Nes-Cre*) and control (*Itpa^fl/fl^*) P16 male mice are shown. Scale bar: 1 cm. (**C**) Postnatal growth delay. Medians of body weight of each genotype group of female (F) and male (M) mice on P0, P8, and P16 are shown. Detailed data of each group and results of their statistical analysis are shown in **D** and Supplemental Figure 4A. (**D**) Body weight on P16. The body weights of control (male: *n* = 37, female: *n* = 52) and *Itpa*-cKO (male: *n* = 15, female: *n* = 16) mice on postnatal day 16 are shown as box plots. Statistical analyses were performed with the Kruskal-Wallis test followed by the Steel-Dwass test for a post hoc comparison. Kruskal-Wallis test, male *P* < 0.0001, and female *P* < 0.0001; Steel-Dwass test, male, *Itpa^fl/fl^/Nes-Cre* (*Itpa*-cKO) vs. *Itpa^+/fl^* ***P = 0.0001, *Itpa*-cKO vs. *Itpa^fl/fl^* ^####^*P* < 0.0001, *Itpa*-cKO vs. *Itpa*******^+/fl^*/Nes-Cre* ****P < 0.0001, Female, *Itpa*^fl***/***fl^*/Nes-Cre* (*Itpa*-cKO) vs. *Itpa^+/fl^* ****P < 0.0001, *Itpa*-cKO vs. *Itpa^fl^* ******^/fl^** ^####^*P* < 0.0001, *Itpa*-cKO vs. *Itpa*******^+/fl^*/Nes-Cre* *P = 0.015. (**E**) Weight ratio of brain to body on P16. The weight ratio of the brain to the body of control and *Itpa*-cKO mice on P16 are shown as box plots. Kruskal-Wallis test, male *P* = 0.1308, and female *P* = 0.0726.

We analyzed the survival of 15 *Itpa*-cKO and 59 control mice for 60 days after birth. During this period, all 15 *Itpa*-cKO mice died within the first 22 days, while all 59 ITPA-proficient control mice survived. A log rank test using a Kaplan-Meier survival curve revealed that the survival of *Itpa*-cKO mice was significantly shorter than that of control mice (Figure 2A). Because the *Itpa-*cKO mice seemed to have smaller bodies than the control mice at P16 (Figure 2B), we measured their body weight and brain weight at P0, P8, and P16. At P0 and P8, the *Itpa-*cKO mice did not show any significant differences in the body weight, brain weight, or ratio thereof compared with mice with 3 other genotypes (Figure 2C and Supplemental Figure 4, A–C). However, the *Itpa*-cKO mice did show a significant reduction in the body weight compared with the other genotypes at P16, indicating severe growth retardation in the *Itpa*-cKO mice (Figure 2, C and D). The *Itpa*-cKO mice were usually debilitated approximately 1 day before their spontaneous early death. Thus, we consider that the majority of deaths were due to failure to thrive. Human ITPA-deficient patients reportedly show microcephaly in addition to growth retardation (3), but no significant difference was noted between the *Itpa*-cKO mice and other control mouse groups in the ratio of brain weight to body weight, even at P16 (Figure 2E and Supplemental Figure 4C).

To determine the reason for the growth retardation and early death of *Itpa*-cKO mice, we performed a biochemical analysis of peripheral blood serum. Significant increases in the blood urea nitrogen (BUN) level (*P* = 0.0086) and decreases in the Ca level (*P* = 0.042) were noted in the serum of *Itpa*-cKO mice compared with control mice. The increased BUN levels may be due to the dehydration of *Itpa*-cKO mice. The reduced Ca level in *Itpa*-cKO mice was quite slight and was not likely the cause of their early death (Supplemental Figure 5). In the present analysis, we were unable to clarify the reason for the growth retardation and early death of the *Itpa*-cKO mice.

### Histopathological analyses.

**Figure 3:**
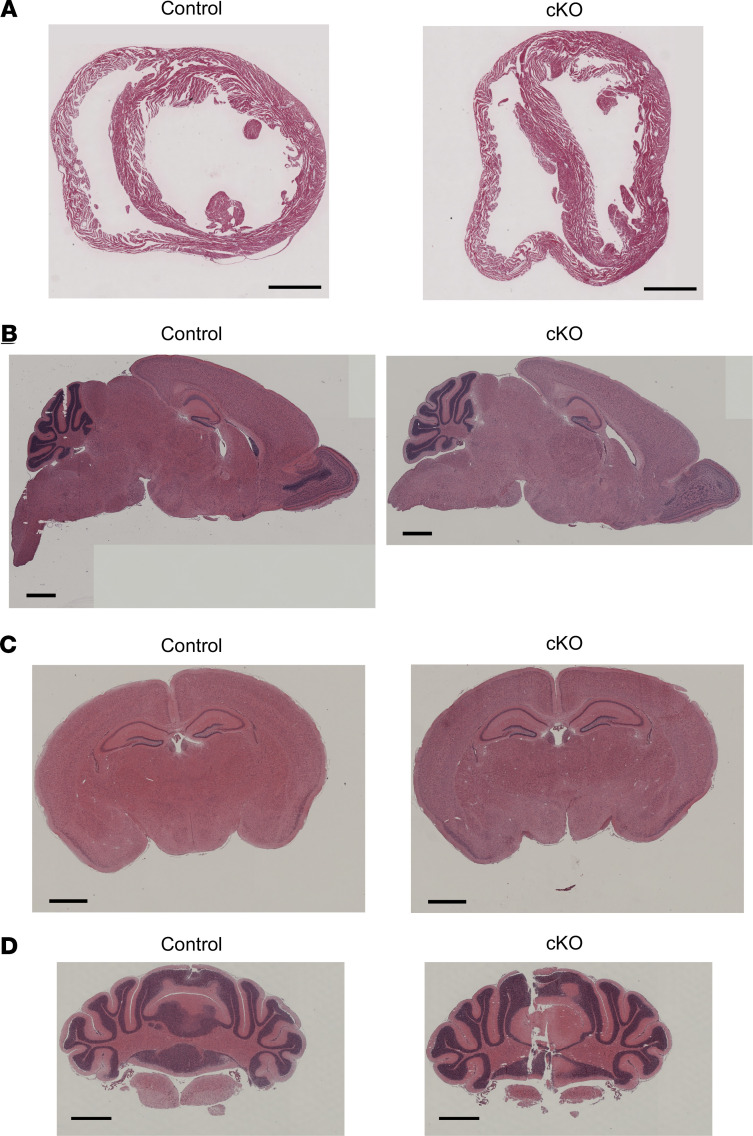
H&E staining of mouse heart and brain sections. (**A**) H&E staining of thin sections of paraffin-embedded heart samples from P16 control (left; *Itpa^fl/fl^*) and *Itpa*-cKO (right) female mice. Scale bars: 500 μm. (**B**–**D**) H&E staining of sagittal thin sections (**B**), coronal thin sections including cerebrum (**C**), and coronal thin sections including cerebellum (**D**) of paraffin-embedded whole brains from P16 control male mice (left; *Itpa^+/fl/^Nes-Cre*) and *Itpa*-cKO male mice (right). Scale bar: 1 mm.

We performed histopathological analyses of paraffin-embedded thin sections of tissues collected from *Itpa*-cKO and control mice at P16 using various staining methods, as shown below. In hematoxylin and eosin (H&E) staining of heart sections of *Itpa*-cKO mice, no abnormalities in the structure of cardiac muscle, including thinning, were observed in systemic *Itpa*-KO mice (Figure 3A) (24). H&E staining of sagittal and coronal thin sections of mouse brains also did not show any apparent histopathological changes, although a more precise analysis may be necessary in order to conclude that no changes whatsoever occurred in *Itpa*-cKO mice (Figure 3, B–D; and Supplemental Figure 6).

**Figure 4:**
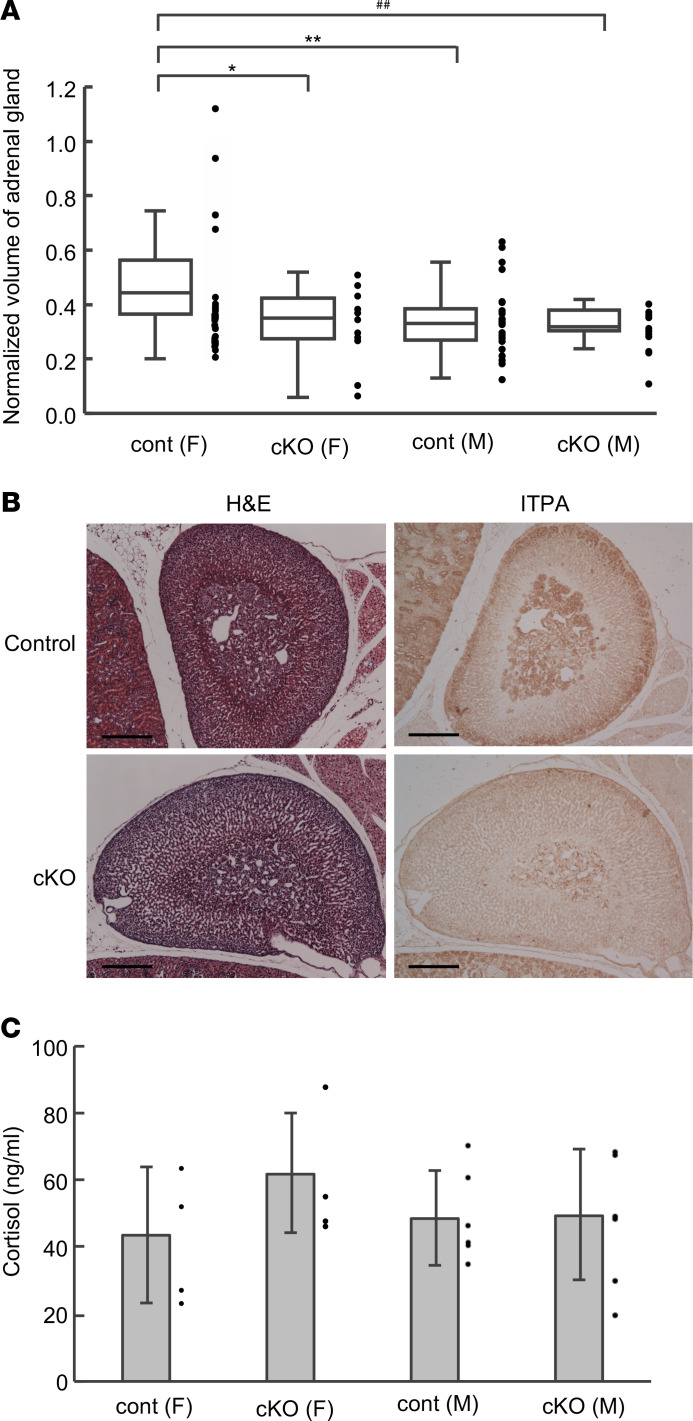
Female-specific hypoplasia of adrenal glands. (**A**) Relative volume of the adrenal gland. The relative volumes of the adrenal glands from P16 *Itpa*-cKO and control male and female mice were calculated as cubes of their longest diameters followed by normalization with their body weights (normalized volume of adrenal gland) and shown as box plots. Statistical analyses were performed with the Kruskal-Wallis test followed by the Steel-Dwass test for a post hoc comparison. Kruskal-Wallis test, *P* = 0.0005; Steel-Dwass test, control female (cont [F][*n* = 36]) vs. *Itpa*-cKO (F) (*n* = 16) *P = 0.0297, cont (F) vs. control male (cont [M] [*n* = 32]) **P = 0.0028, cont (F) vs. *Itpa*-cKO (M) (*n* = 16) ^##^*P* = 0.0056. (**B**) H&E staining and ITPA immunohistochemistry of the adrenal glands. Two adjacent thin sections of paraffin-embedded adrenal glands from P16 control (upper) and *Itpa*-cKO (lower) female mice were subjected to H&E staining (left) and immunohistochemistry with anti-ITPA. Scale bar: 200 μm. (**C**) Cortisol content in mouse serum. Serum samples were prepared from P16 control and *Itpa*-cKO male and female mice and analyzed for their cortisol contents using DetectX Cortisol Enzyme Immunoassay Kit (Arbor Assays, Ann Arbor, Michigan, USA). Statistical analyses were performed with a 1-way ANOVA, *P* = 0.517.

Regarding our findings in tissues other than the brain, the adrenal glands of female *Itpa*-cKO mice showed hypoplasia (Figure 4A). In the quantitative analysis of hypoplasia, we measured the longest diameter of the adrenal glands of *Itpa*-cKO and control mice. The cube of the longest diameter was considered to reflect the relative volume of each adrenal gland, and the relative volume of the adrenal gland per gram of body weight (normalized volume of the adrenal gland) in control female mice was higher than that in control male mice, as found in previous reports (27, 28). The normalized volume of the adrenal gland was significantly decreased in *Itpa*-cKO female compared with control female mice, but no marked difference was observed between *Itpa*-cKO and control male mice. We also analyzed the ITPA expression by immunohistochemistry with anti-ITPA and detected ITPA signals in the outer area of the adrenal cortex and adrenal medulla of control female mice. These signals were found to be attenuated in the adrenal glands of *Itpa-*cKO female mice (Figure 4B). To analyze the biological function of the adrenal glands, we measured the peripheral serum level of cortisol, which is produced by the adrenal cortex. No significant difference in the cortisol content was noted between *Itpa*-cKO and control mice (Figure 4C).

### Tail suspension test.

**Figure 5:**
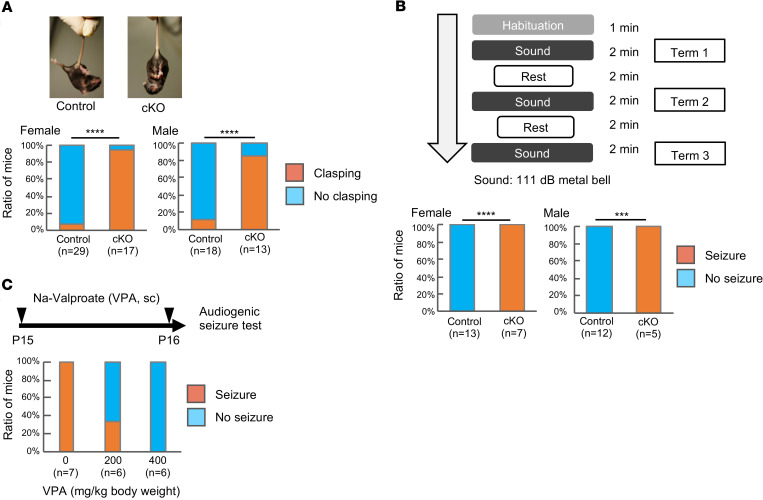
Behavior analyses of *Itpa*-cKO mice. (**A**) Limb-clasping in the tail suspension test. P16 *Itpa*-cKO female mice (right) but not control female mice (*Itpa^fl/fl^*) (left) showed limb-clasping behavior in the tail suspension test. The summarized results of female and male mice are shown as component bar charts. Statistical analyses were performed with Fisher’s exact test. Female control vs. female *Itpa*-cKO ****P < 0.0001, male control vs. male *Itpa*-cKO ****P < 0.0001. (**B**) Audiogenic seizure induction test. Each P16 mouse was adapted in an empty plastic cage for 1 minute and then exposed to audio stress by a metal bell for 2 minutes 3 times. The mouse rested for 2 minutes between the audio stress exposures. The summarized results of female and male mice are shown as component bar charts. Statistical analyses were performed with Fisher’s exact test. Female control vs. female *Itpa*-cKO ****P < 0.0001, male control vs. male *Itpa*-cKO *P* = ***0.0002. (Genotypes of the controls are shown in Supplemental Table 2.) (**C**) Suppression of audiogenic seizure by sodium valproate treatment. Each *Itpa*-cKO male mouse was injected with sodium valproate (sc, 200 or 400 mg/kg body weight) or vehicle on P15 and P16 and then subjected to the audiogenic seizure test 1 hour later. The summarized results are shown as a component bar chart. Statistical analysis was performed with Cochran-Armitage trend test, *P* = 0.0003.

We noticed the limb flexion of the *Itpa*-cKO mice during the cage exchange process. Some mutant mice with brain pathologies show limb flexion instead of the limb extension observed in normal mice when they are picked up by the tail (tail suspension test) (29). This behavior is called paw-clasping or limb-clasping and is regarded as a symptom of neurological deterioration. Therefore, we performed the tail suspension test in our mice, and limb-clasping was observed significantly more often in *Itpa*-cKO mice than in ITPA-proficient mice (*P* < 0.0001 in both male and female mice) (Figure 5A).

### Spontaneous and audiogenic seizures.

We did not perform 24-hour monitoring of mouse behavior because the *Itpa*-cKO mice tended to die before weaning. However, we found that at least 3 of the 100 *Itpa*-cKO female mice and 2 out of the 114 male mice exhibited spontaneous and generalized tonic-clonic seizures with wild running (Supplemental Video 1). The spontaneous seizures in the 5 mice were sustained for a short period and did not result in immediate death. Then, we wanted to evaluate the susceptibility of the *Itpa*-cKO mice to seizures in the absence of any pharmacological or electric component. Mice differ from humans in that they are generally susceptible to audiogenic seizures (30–32). Although the C57BL/6 mouse is relatively resistant to audiogenic seizures, acoustic priming or some genetic modifications are known to induce audiogenic seizures even in mice with a C57BL/6 genetic background (33). Thus, we performed an audiogenic seizure induction test by exposing mice to metal bell sounds (110–112 db). All 7 female *Itpa-*cKO and 5 male *Itpa*-cKO mice showed generalized tonic-clonic seizure during the exposure, whereas ITPA-proficient mice did not show any seizure (Supplemental Video 2, Figure 5B, and Supplemental Table 2). *Itpa*-cKO mice were significantly more susceptible to audiogenic seizure than ITPA-proficient mice (*P* < 0.0001 in female mice; *P* = 0.0001 in male mice). When we did not stop the audio stress, the audiogenic seizure resulted in sudden unexpected death in epilepsy (SUDEP) in the *Itpa-*cKO mice in contrast to spontaneous seizures without SUDEP.

To assess the potential application of the *Itpa*-cKO mouse as an animal model of human ITPA deficiency, we administered sodium valproate, a broad-spectrum anticonvulsant that inhibits GABA transaminase, voltage-gated sodium channel, voltage-gated calcium channel, and histone deacetylases (34), to *Itpa*-cKO mice because sodium valproate is one of the major medications used to treat human generalized seizures and infrequent seizures of one patient with ITPA deficiency were reported to be well controlled by antiepileptic medication (20). We administered vehicle (normal saline) or sodium valproate to *Itpa*-cKO mice (200 or 400 mg/kg/d, subcutaneous [sc]) for 2 days and performed the audiogenic seizure induction test at 1 hour after the final administration. All *Itpa*-cKO mice administered vehicle showed generalized seizure, as did naive *Itpa*-cKO mice, whereas only 33.3% and 0% of mice administered sodium valproate at a dose of 200 or 400 mg/kg/d showed seizure, respectively. This finding shows significant and dose-dependent suppression of audiogenic seizures in *Itpa*-cKO mice by sodium valproate (*P* < 0.003) (Figure 5C).

### Electrophysiological analyses of mouse brains.

The resting membrane potential and miniature excitatory postsynaptic current (mEPSC) are reportedly often altered in the neurons of mouse models of epileptic seizure (35–37). To clarify why ITPA deficiency resulted in generalized seizure, we performed an electrophysiological analysis of entorhinal pyramidal cells in layers II/III of brain slices from our mice, as this region serves as the major interface between the hippocampus and sensory cortical regions.

**Figure 6:**
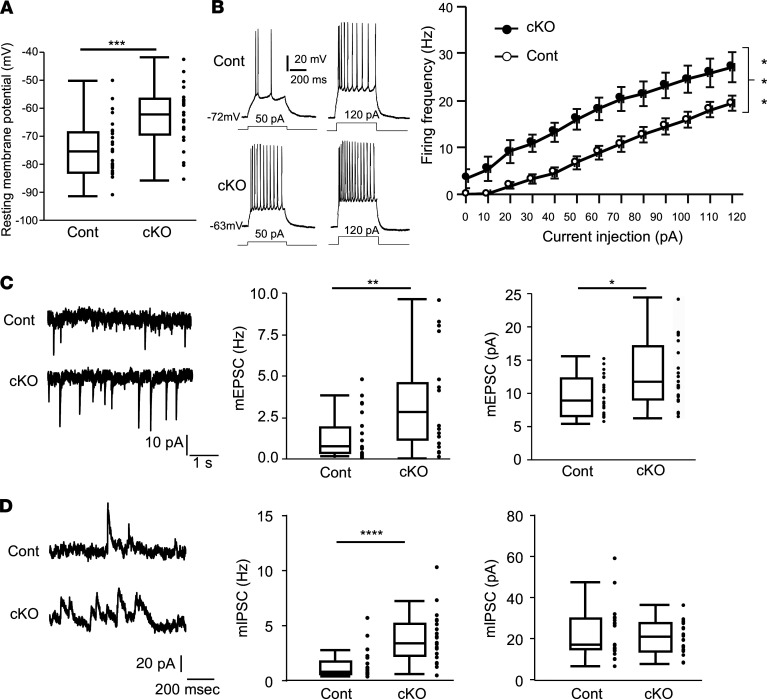
Electrophysiological properties of entorhinal cortex neurons of P15 to P18 male mice. (**A**) Resting membrane potential (RMP). The RMP of entorhinal cortex neurons was analyzed by the whole-cell current-clamp test with sliced mouse brains and shown as box plots. Control cells (26 cells from 3 male control mice) and *Itpa*-cKO cells (24 cells from 3 male *Itpa*-cKO mice) were analyzed. Statistical analyses were performed with Wilcoxon’s rank sum test; ***P = 0.0005. (**B**) Action potential (AP) firing. AP firings of *Itpa*-cKO cells (21 cells from 3 *Itpa*-cKO male mice) and control cells (25 cells from 3 control male mice; *Itpa^fl/fl^*) were detected with or without current injection. The current injection-dependent increase in the frequencies of AP firings is shown as the mean ± SD (right). Representative recordings of AP firing from control (upper) and *Itpa*-cKO (lower) brain slices are shown on the left. A 2-way repeated measures ANOVA using least square regression, *Itpa*-cKO vs. control ***P = 0.0008. (**C**) Miniature excitatory postsynaptic current (mEPSC). mEPSCs of *Itpa*-cKO cells (21 cells from 3 *Itpa*-cKO male mice) and control cells (24 cells from 3 control male mice) were analyzed by voltage-clamp recordings. Representative trace (left) and box plots of the frequency (middle) and amplitude (right) of mEPSCs are shown. Statistical analyses were performed with Wilcoxon’s rank sum test, frequency **P = 0.005, amplitude *P = 0.0209. (**D**) Miniature inhibitory postsynaptic current (mIPSC). mIPSCs of *Itpa*-cKO cells (23 cells from 3 *Itpa*-cKO male mice) and control cells (21 cells from 3 control male mice) were analyzed. Representative trace (left) and box plots of the frequency (middle) and amplitude (right) of mIPSCs are shown. Statistical analyses were performed with Wilcoxon’s rank sum test, frequency ****P < 0.0001, amplitude *P* = 0.7069.

Whole-cell patch-clamp recordings in brain slices of *Itpa*-cKO and *Itpa^fl/fl^* male and female mice (control) from 15 to 18 days of age revealed the statistically significant depolarization of the resting membrane potential in the entorhinal pyramidal cells of *Itpa*-cKO mice compared with control mice (Figure 6A and Supplemental Figure 7). In addition, the action potential firing frequency in the entorhinal pyramidal cells of *Itpa*-cKO male mice was significantly higher than in control male mice in the analysis with current pulses injection up to 120 pA (Figure 6B). The basal firing activity in entorhinal pyramidal cells was also increased in *Itpa*-cKO mice (Figure 6B).

To further explore the enhanced neuronal activity in *Itpa*-cKO mice, we analyzed the synaptic activities. The frequency of both mEPSC and miniature inhibitory postsynaptic current (mIPSC) in *Itpa*-cKO pyramidal cells was significantly higher than in control cells (mEPSC: *P* < 0.01; mIPSC: *P* < 0.001) (Figure 6C, middle, and Figure 6D, middle). The amplitude of mEPSC in *Itpa*-cKO cells was also significantly higher than in control cells, although no significant difference was noted in the amplitude of mIPSC between *Itpa*-cKO cells and control cells (Figure 6C, right, and Figure 6D, right). Seizure-like events were recently reported to induce the potentiation of α-amino-3-hydroxy-5-methyl-4-isoxazolepropionic acid receptor in pyramidal cells of rat entorhinal cortex (37). The increase in the amplitude of mEPSC in the entorhinal pyramidal cells of *Itpa*-cKO mice may also depend on the increase in presynaptic excitation observed as the frequency of mEPSC increases.

## Discussion

Neural stem cell–specific *Itpa*-cKO mice, which we generated in this study, showed growth retardation after the first 8 days of their lives and died in the early postnatal period. Most *Itpa*-cKO mice died from 18 to 22 days after birth, while conventional *Itpa*-KO mice, which we previously established, died within 2 weeks after birth (24). *Itpa*-KO but not *Itpa*-cKO mice show embryonic lethality and ventricular wall thinning (24). These differences between the 2 mouse lines seem to depend on the remaining ITPA expression in the cells that did not originate from neural stem cells in *Itpa*-cKO mice. *Itpa*-cKO mice died in the very short period around weaning. Some other genetic mouse models of severe epilepsy also show early death around weaning (38–40). It might be associated with the susceptibility of young mice (P20 to P25) to seizures, as reported in audiogenic seizure mouse strains (33, 41, 42), although the death of *Itpa*-cKO mice does not seem to be SUDEP.

The present study showed that ITPA deficiency in the neural system induces growth retardation and early death. This growth retardation may be due to poor nutrition caused by digestion; absorption disorder owing to an abnormality in the neural system innervating the gastrointestinal smooth muscles; or feeding disability resulting from cerebellar ataxia, although the data from blood biochemical tests did not suggest poor nutrition. Dysfunction in the production or secretion of hormones in endocrine tissues associated with neural stem cells, such as the pituitary gland and adrenal medulla, may also cause growth retardation (43). Further studies will be needed in order to elucidate the mechanism underlying the growth retardation and early death observed in this model.

Ten cases of human ITPA deficiency have previously been described (3, 20, 21). All 10 patients presented with developmental delay, microcephaly, and seizure, while some additionally showed dilated cardiomyopathy. Eight of the 10 patients were reported as having early infantile-onset epileptic encephalopathy (3, 20), while the remaining 2 patients were reported to have Martsolf-like syndrome with lethal infantile dilated cardiomyopathy (21). Most died from heart failure, status epilepticus, or respiratory infection by 4 years of age. The *Itpa*-cKO mice in the present study showed growth retardation and spontaneous and audiogenic seizure and died in the early postnatal period, but they did not show microcephaly. The lack of microcephaly in our *Itpa*-cKO mice may be due to species differences.

Electrophysiological analyses of brain slices of *Itpa*-cKO mice showed significant depolarization of the resting membrane potential and a significant increase in the action potential firing frequency with or without pulse current injection in entorhinal pyramidal cells, in comparison with control mice (Figure 6, A and B). Several potential causes of the resting membrane potential depolarization of the neurons in *Itpa*-cKO mice are proposed, such as the deterioration of the Na^+^/K^+^-ATPase function (Supplemental Figure 8) and a change in the membrane permeability for Na^+^, K^+^, or Cl^–^ via ion channels. Because of its molecular structure, which is similar to that of ATP, ITP can act as an aberrant substrate replacing ATP in some biological processes. For example, it has been shown that Mg·ITP-bound actomyosin has a greatly reduced shortening velocity and rate of force recovery in comparison with the Mg·ATP-bound form and shows disordered striations during activation in vitro (44), which is a hypothetical mechanism of abnormal cardiac development in ITPA deficiency (24). In the same way, the Na^+^/K^+^-ATPase of neural cells might be inhibited by accumulated ITP, resulting in the depolarization of the resting membrane potential. The ATP-sensitive K^+^ channel is a leaky K^+^ channel that maintains a low resting membrane potential by excluding intracellular K^+^ ions. ITP may alter the opening of this K^+^ channel. Another potential cause is a change in the extracellular environment caused by ITPA-deficient astrocytes, which regulate neuronal excitability by maintaining the extracellular glutamate, GABA, and K^+^ levels (45–47). For example, the absence of K^+^ ion buffering via Kir4.1 or the glutamate uptake by Glt-1 or GLAST on astrocytes has been reported to cause extracellular K^+^ or glutamate accumulation after repeated neuron firing (48, 49). However, the targeted disruption of the *Kir4.1* gene in mouse astrocytes increased neither the resting membrane potential nor the action potential frequency of CA1 pyramidal neurons (50), and the in vivo knockdown of *Glt-1* or *Glast* induced a significant decrease in the mEPSC in mouse layer V pyramidal neurons, in contrast to those in the entorhinal cortex pyramidal neurons of *Itpa*-cKO mice (51). The increase of mIPSC frequency in the entorhinal pyramidal cells of *Itpa*-cKO mice indicates that inhibitory neurons, including interneurons, were excited more frequently, suggesting that the neural hyperexcitation in *Itpa*-cKO mice is not caused by disinhibition.

As discussed in previous reports (21, 24), accumulated ITP/dITP may induce various biological changes via direct mechanisms, such as competition with ATP/dATP or GTP/dGTP, or indirect mechanisms through the generation of their metabolites, such as deoxyinosine-containing DNA or inosine-containing RNA. Cyclic inosine monophosphate also can be generated from ITP by guanylate cyclase (52), which may depolarize the resting membrane potential by opening cyclic nucleotide-gated ion channels (53, 54). Whether or not such neuronal excitability can occur in humans with a low ITPA activity is important to determine. Humans homozygous for the SNP of *ITPA* (rs1127354, 94C>A) have been reported to have reduced ITPA activity and show an abnormal ITP accumulation in their erythrocytes (17). Furthermore, another *ITPA* SNP (rs6084309) was recently reported to be significantly associated with general risk tolerance as well as SNPs related to glutamatergic or GABAergic neurotransmissions in a multitrait analysis of genome-wide association studies (55). These *ITPA* SNPs may influence normal human behavior or cause abnormal neuronal excitability in some genetic backgrounds or pathological situations through the depolarization-dependent mechanism shown in our data.

In the present study, we performed histopathological analyses of several tissues other than brain and recognized adrenal hypoplasia in *Itpa*-cKO mice. In the adrenal glands of *Itpa*-cKO mice, the cells were sparse, particularly in the cortex. Nestin-positive adrenocortical progenitors, which exist between the zona glomerulosa and zona fasciculata, have been reported to supply cortical cells to the adrenal cortex in the postnatal period (56). In these precursor cells in the *Itpa*-cKO mice, the deletion of *Itpa* exon 5 may have occurred and resulted in a decreased supply of newly differentiated cells. Indeed, the ITPA signals in this region were lower in *Itpa*-cKO mice than in control mice. The female specificity of adrenal hypoplasia in *Itpa*-cKO mice may depend on the female-specific increased adrenal formation in control mice.

We were able to suppress audiogenic seizure of *Itpa*-cKO mice through sodium valproate administration in our study. This indicates that *Itpa*-cKO mice are a good model for evaluating the therapies of ITPA deficiency. Sodium valproate is used to control seizures of infant patients with West syndrome, early-onset epileptic encephalopathy, or early infantile epileptic encephalopathy. As ITPA deficiency causes not only neural system disorder but also cardiac abnormalities in mice and humans, the treatment of ITPA-deficient patients with only antiepileptic drugs, such as sodium valproate, is doubtless inadequate; drugs that can suppresses ITP accumulation are likely to be more effective for treating ITPA deficiency. Our *Itpa-flox* mice were found to be useful tools for identifying and evaluating such drugs, respectively, although a comparison of their effects on our models and other epilepsy models will be necessary in the future to identify their specificities.

In conclusion, the present study showed that ITPA deficiency causes depolarization of the resting membrane potential and a high frequency of excitation of neurons, which may be the cause of the observed seizures. We hope that further studies will clarify the relevant mechanism in detail and encourage the development of new treatments for ITPA deficiency.

## Methods

### Animal care.

All animals were maintained in a temperature-controlled (22°C ± 2°C, 55% ± 5% humidity), specific pathogen–free room with a 12-hour light/12-hour dark cycle.

### Oligo DNA primers.

All oligo DNA primers used for sequencing and polymerase chain reaction (PCR) are listed in Supplemental Table 1.

### Establishment of Itpa^+/fl^ mice.

To generate tissue-specific *Itpa*-KO mice, we first established a mouse line with a floxed mouse *Itpa* allele, in which exon 5 is flanked by 2 *loxP* sites, as shown below. From the NIH Knock-Out Mouse Program (KOMP, Bethesda, Maryland, USA), we obtained a targeting vector (PRPGS00067_A_H05) with floxed exon 5 of mouse *Itpa* genomic DNA (Supplemental Figure 1A) that had been designed to generate the *Itpa*^tm2a(EUCOMM)Wtsi^ allele. We then analyzed its base sequence by Sanger sequencing with the sequencing primers in Supplemental Table 1. The results showed an unexpected deletion of 2380 bp, including exons 2, 3, and 4. The presence of highly homologous sequences on both the 5′ and 3′ termini of this region suggested that homologous recombination may have excised this region during vector construction in *Escherichia coli*. This deletion made it a targeting vector for nonconditional gene disruption.

In addition, there was a transposon 10–like 1331 bp insertion in the *LacZ* coding sequence of the promoter-driven cassette L1L2_Bact_P cassette. Therefore, we reconstructed the targeting vector by returning exons 2, 3, and 4. We did not remove the transposon-like insertion because it would be excised with the L1L2_Bact_P cassette by FLPe recombinase at a later step.

First, we amplified the 3566 bp DNA fragment containing exons 2, 3, and 4 but not the 3′-homologous regions by PCR using the primer set ItpaEx234Fw3/ItpaEx234Rv3 (Supplemental Table 1) and genomic DNA from JM8A3, an embryonic stem (ES) cell line with a C57BL/6N genetic background (KOMP), as a template. A 3560 bp fragment was prepared from the PCR product by *Asi*SI/*Afl*II restriction enzyme treatment and exchanged with a 2193 bp fragment flanked by the *Asi*SI/*Afl*II sites in the vector PRPGS0067_A_H05, resulting in the generation of the new targeting vector without exon deletion. The base sequence of the new vector was confirmed by Sanger sequencing. The structures of the original and reconstructed new targeting vectors are shown in Supplemental Figure 1A.

The new targeting vector was linearized by *Pac*I digestion and purified by phenol/chloroform treatment and ethanol precipitation. The vector (5 μg) was transferred into the ES cell line JM8A3 using Microporator (Digital Bio Technology, Seoul, South Korea). The cells were then cultured on a feeder cell layer in ES medium (KnockOut DMEM from Thermo Fisher Scientific Inc., Sunnyvale, California, USA; 15% fetal bovine serum; 1 mM nonessential amino acids; 2 mM GlutaMax from Thermo Fisher Scientific Inc.; 0.1 mM 2-mercaptoethanol; and 1 × 10^3^ U/mL of recombinant mouse leukemia inhibitory factor from Merck, Darmstadt, Germany). The feeder cell layer was composed of mouse embryonic fibroblasts treated with mitomycin C (MilliporeSigma Japan, Tokyo, Japan). The next day, 100 μg/mL of G418 (MilliporeSigma Japan) was added to the culture for selection of stably transfected clones. The medium was exchanged every day. After 7 days’ culture, colonies were isolated and expanded in the presence of 50 μg/mL of G418. The presence of the targeted allele (the LacZ/neo allele) in each clone was confirmed by genotyping PCR with the primer sets Itpa-GF3/5′Universal(LAR3) for the 5′ 5112 bp of the LacZ/neo allele, 3′Universal(RAF5)/Itpa-GR4 for the 3′ 6442 bp of the LacZ/neo allele, 3rdloxPFw/Itpa-GR4 for the 3′ *loxP* site-specific 5538 bp of the LacZ/neo allele, and Itpa-GF3/Itpa-ex4Rv for the common 4328 bp of the wild LacZ/neo and floxed *Itpa* alleles (Supplemental Table 1). This LacZ/neo *Itpa* allele (Supplemental Figure 1A) was registered as *Itpa*^tm1Yun^ in Mouse Genome Informatics (MGI: 6305062) and the DNA Data Bank of Japan (DDBJ accession LC484010).

ES clone 40 carrying the LacZ/neo allele was injected into the blastocysts of C57BL/6J mice (CLEA Japan, Inc., Tokyo, Japan) and transferred into female ICR mice (CLEA Japan, Inc.) to obtain chimeric mice. Male chimeric mice were mated with female C57BL/6J mice to obtain F1 mice. Female F1 mice with the LacZ/neo *Itpa* allele were then selected via genomic PCR with the primer set Fw1afterFLP/5′Universal(LAR3) (Supplemental Figure 1C) and mated with male *CAG*-*FLPe* deleter mice with a C57BL/6J genetic background [RBRC01834 B6-Tg(CAG-FLPe)36; RIKEN BioResource Center, Japan] (57) to excise the Promoter-Driven Cassette L1L2_Bact_P cassette flanked with FRT sequences. N2 mice with floxed *Itpa* allele without the Promoter-Driven Cassette L1L2_Bact_P cassette (floxed allele in Supplemental Figure 1A), which was registered as *Itpa*^tm1.1Yun^ in Mouse Genome Informatics (MGI: 6305063) and the DNA Data Bank of Japan (DDBJ accession LC484011), were selected by genomic PCR with the floxed-allele-specific primer set Fw2afterFLP/Rv2afterFLP (223 bp) and the neo-cassette-specific primer set NER1/NEL2 (450 bp) (Supplemental Figure 1C). WT *Itpa* alleles were detected by genomic PCR with the specific primer set Itpa-5′/Itpa-3′ (product size: 590 bp) as a control. The mice with the *Itpa*^tm1.1Yun^ allele (*Itpa^fl^* allele) were then mated with C57BL/6J mice to exclude *CAG-FLPe* transgene. N3 mice with *Itpa*^tm1.1Yun^ allele and without *CAG-FLPe* transgene were used for further mating as *Itpa^+/fl^* mice. The *CAG-FLPe* transgene was detected by genotyping PCR with the specific primer set FLP/Flp-IntR (product size: 372 bp) and the internal control primer set IMR0015/IMR0016 (product size: 210 bp).

### Preparation of Itpa^fl/fl^/Nes-Cre mice.

To prepare neural stem cell–specific *Itpa*-KO mice, we used B6.Cg-Tg(Nes-cre)1Kln/J mouse (*Nes-Cre*; The Jackson Laboratory 003771, C57BL/6J background; The Jackson Laboratory, Bar Harbor, Maine, USA), in which the rat *Nestin* promoter-driven *Cre*-recombinase gene is expressed. First, N3 *Itpa^+/fl^* mice were inbred or cross-mated with hemizygous *Nes-Cre* mice to generate *Itpa^fl/fl^* and *Itpa^+/fl^/Nes-Cre* mice, respectively. The *Itpa^fl/fl^* and *Itpa^+/fl^/Nes-Cre* mice were then cross-mated to generate *Itpa^fl/fl^/Nes-Cre* neural stem cell–specific *Itpa*-KO mice with a hybrid genetic background of C57BL/6J and C57BL/6N. The detection of WT *Itpa* allele without *loxP* sites and *Cre*-recombinase transgene was performed by PCR using the primer sets Itpa-5′/Itpa-3′ and Cre-1/Cre-2 (product size: 260 bp), respectively.

### Genotyping PCR.

Genotypes were determined by a PCR analysis of genomic DNA samples derived from mouse tails or cultured ES cells using Mighty Amp DNA polymerase (Takara Bio Inc., Shiga, Japan). The primer sequences used for the genotyping are shown in Supplemental Table 1.

### Tissue processing.

For RNA extraction, 16-day-old mice were euthanized by cervical dislocation and then quickly dissected. The obtained tissues were snap-frozen in liquid nitrogen and preserved at –80°C. For the Western blot analysis, 16-day-old mice were anesthetized with the combination of 3 anesthetics: medetomidine hydrochloride (Domitol; Meiji Seika Pharma Co., Ltd., Tokyo, Japan) 0.3 mg/kg; midazolam (Dormicum; Astellas Pharma Inc., Tokyo, Japan) 4 mg/kg; and butorphanol (Vetorphale; Meiji Seika Pharma Co., Ltd.) 5 mg/kg (58), then transcardially perfused with normal saline and quickly dissected. The obtained tissues were snap-frozen in liquid nitrogen and preserved at –80°C.

For histopathological analyses, 16-day-old mice were anesthetized with the combination of 3 anesthetics and transcardially perfused with normal saline, followed by 4% paraformaldehyde (PFA). Tissues were quickly dissected and immersed for 24 hours in 4% PFA. After PFA treatment, the brain samples were further immersed for 24 hours in 20% sucrose and for another 24 hours in 30% sucrose at 4°C. All tissue samples were then stored as paraffin-embedded blocks.

### Partial purification of anti-ITPA rabbit immunoglobulin.

The immunoglobulin (Ig) fraction was precipitated from rabbit antiserum against the TrxA-human ITPA fusion protein (13) by ammonium sulfate precipitation as described previously (59) and partially purified by eliminating antibodies that bind to the carboxyl terminal of mouse ITPA (mITPA-C) fused to bacterial thioredoxin A (TrxA-mITPA-C). A DNA fragment coding mITPA-C was amplified from pET32a:mITPA (13) by PCR with a primer set mITPA_C Nco_beta4_Fw#2/mITPA_C_Hind_Rv (Supplemental Table 1). The PCR product was cloned into *Hind*III/*Nco*I site of pET32a expression vector (Novagen, Merck) to generate pET32a:mITPA-C. TrxA-mITPA-C protein was then prepared, immobilized on NHS-activated Sepharose 4 Fast Flow Beads (GE Healthcare, Chicago, Illinois, USA), and used to eliminate binding proteins from the Ig fraction of anti-ITPA serum, as described previously (59). The TrxA-mITPA-C–unbound Ig fraction from anti-ITPA serum was used as partially purified anti–ITPA-N antibody.

### Western blotting.

Frozen tissue samples from 3 control male mice (*Itpa^fl/fl^*) and 3 *Itpa*-cKO male mice (age, P16 or P17) were homogenized in 1× sodium dodecyl sulfate (SDS) sample buffer (62.5 mM Tris-HCl pH 6.8, 2% SDS, 5% glycerol, 2% 2-mercaptoethanol, and 0.005% bromophenol blue) using a potter Teflon homogenizer (Thomas Scientific, Swedesboro, New Jersey, USA) at 4°C. Their protein concentrations were analyzed by using an XL-Bradford (SDS-PAGE) reagent (Aproscience, Tokushima, Japan). Denatured protein samples (5 μg of total protein/lane) were separated by SDS-PAGE and transferred onto an Immobilon-P PVDF membrane (Merck, Darmstadt, Germany). Blocking of the membranes was performed by incubation for 1 hour at room temperature in Tris-buffered saline with Tween-20 (TBST; 10 mM Tris-HCl pH 7.5, 0.9% NaCl, 0.1% Tween-20) containing 5% nonfat dried milk (Megmilk snow brand, Tokyo, Japan). Each membrane was separated into 2 parts: an upper part containing proteins larger than 25 kDa and a lower part containing proteins smaller than 25 kDa. The lower parts were incubated in TBST containing anti-ITPA rabbit antiserum (13, 60) (1:2000 dilution) for 16 hours at 4°C, with gentle shaking. The upper parts were incubated in TBST containing anti-GAPDH antibody (MAB374, Merck, Darmstadt, Germany) (1:100,000 dilution) to detect GAPDH as an internal control protein. The lower and upper membranes were then washed with TBST and incubated in TBST containing anti–rabbit IgG HRP-linked goat antibody (1:3000 dilution; Cell Signaling Technology, Inc., Danvers, Massachusetts, USA) or anti–mouse IgG HRP-linked goat antibody (1:3000 dilution; Cell Signaling Technology, Inc.), respectively, for 1 hour at room temperature. After washing with TBST, the lower and upper parts from each membrane were set next to each other, and the antibodies that were able to bind to the blots were detected by the chemiluminescence method with the Western BLoT Quant HRP Substrate (Takara Bio Inc.). Digitized images were obtained with an AE-9300 Ez-CaptureMG (ATTO, Tokyo, Japan). Images were analyzed by the densitograph software program CS Analyzer 3 (ATTO). The relative signal intensity of ITPA or GAPDH in each lane was quantified using cerebrum extract from 1 control mouse as a standard sample, different amounts of which were loaded into 5 lanes of each blot.

### Pathological analyses.

For immunohistochemical staining, 4 μm paraffin-embedded sections were deparaffinized with xylene and washed in ethanol. The slides were incubated with 0.3% H_2_O_2_ solution (diluted in distilled water) for 10 minutes to quench endogenous peroxidase activity. After rinsing in phosphate‑buffered saline (PBS; 137 mM NaCl, 2.7 mM KCl, 8.1 mM Na_2_HPO_4_ solution, pH 7.6), the sections were blocked in 1× Block Ace solution (Dainippon Pharmaceutical, Osaka, Japan) at room temperature for 1 hour and incubated overnight at 4°C in the partially purified rabbit anti–ITPA-N antibody (0.5 μg/mL in PBS). After rinsing in PBS, the sections were incubated with a biotinylated goat anti–rabbit IgG antibody (VECTOR Laboratories, Burlingame, California, USA) at room temperature for 45 minutes. VECTASTAIN Elite ABC Standard Kit (VECTOR Laboratories) and DAB Substrate Kit (VECTOR Laboratories) were then used to visualize the bound secondary antibody. Digital images were acquired using an Axio Imager A1 microscope, equipped with an AxioCam charge-coupled device camera and the AxioVision 4.9 imaging software program (Carl Zeiss Microscopy, Tokyo, Japan). Views of entire coronal sections were obtained using a Nikon Eclipse 80i microscope with a Virtual slice module in the Stereo Investigator software program (MBF Bioscience, Williston, Vermont, USA).

### Audiogenic seizure induction tests.

Each 16-day-old mouse was placed in an empty clear plastic cage (25 cm long, 14 cm wide, and 12 cm deep) and allowed to explore the cage for 1 minute. After this habituation period, the mice were exposed to 111 dB metal bell sounds for 2 minutes 3 times with 2-minute intervals between the stimulation periods until generalized seizure was observed. In the sodium valproate administration test, we subcutaneously injected 50 or 100 mg/mL of sodium valproate (MilliporeSigma Japan) in normal saline at 200 or 400 mg/kg body weight at P15 and P16. One hour after the second injection, we performed the audiogenic seizure induction test as described above. After the audiogenic seizure induction tests, the mice were subjected to tissue processing as described above.

### Quantification of riboinosine in RNA by liquid chromatography–tandem mass spectrometry.

Total RNA samples that did not contain any small RNA molecules, such as tRNA, were prepared using the RNeasy Lipid Tissue Mini Kit (QIAGEN Inc., Valencia, California, USA) according to the manufacturer’s instructions in the presence of 100 μM deferoxamine (MilliporeSigma Japan), 100 μM butylated hydroxytoluene (MilliporeSigma Japan), and 20 μM deoxycoformycin (Santa Cruz Biotechnology, Santa Cruz, California, USA). Each RNA sample (27 μg) was supplemented with 250 femtomole of [^13^C, ^15^N]-labeled inosine as internal control, which was prepared from [^13^C, ^15^N]-labeled adenosine (Silantes GmbH, Munich, Germany) by deamination as described previously (61) and digested with nuclease P1 (Yamasa, Chiba, Japan) and alkaline phosphatase (P-5521; MilliporeSigma Japan) to ribonucleosides and monophosphates, as described previously (25). A liquid chromatography–tandem mass spectrometry analysis of inosine in the digested RNA samples was performed using a Nexera X2 LC system (Shimadzu, Kyoto, Japan) connected to a triple-quadrupole mass spectrometer API3200 (AB SCIEX, Framingham, Massachusetts, USA). The digested RNA samples were then applied to an Acclaim Polar Advantage C16 column (3 μm, 3.0 mm × 250 mm; Thermo Fisher Scientific Inc.) maintained at 25°C and eluted at a flow rate 0.2 mL/min using a mobile phase buffer (0.1% acetic acid). The eluent was monitored at 254 nm using an SPD-20A UV/Vis detector (Shimadzu). The amounts of guanosine and adenosine were calculated based on the absorbance of known amounts of standard nucleosides.

The mass spectrometric analysis was carried out in the positive ionization mode with a turbo ion spray source using nitrogen gas as a nebulizer and curtain gas. The mass spectrometer parameters optimized for inosine were as follows: curtain gas: 10 psi; collision gas: 3; ion spray voltage: 5500 V; temperature: 500°C; ion source gas 1: 50 psi; ion source gas 2: 30 psi; declustering potential: 21 V; entrance potential: 5 V; collision energy: 35 V; collision cell exit potential: 3 V. Data were obtained in multiple reaction monitoring mode, using transitions of *m/z* 269 to 137 for inosine and *m/z* 283 to 146 for [^13^C, ^15^N]-labeled inosine. Data acquisition and quantification were performed using the Analyst 1.6.2 software program (AB SCIEX). Each sample was analyzed twice, and the mean of the repeated analyses was regarded as the inosine content in each sample.

### Patch-clamp analyses.

All mice were anesthetized with the combination of 3 reagents as described above and euthanized by decapitation. Entorhinal cortex slices were cut to 200 μm thickness with a VT1000 vibratome (Leica) using ice-cold cutting solution consisting of (in mM) 234 sucrose, 2.5 KCl, 1.25 NaH_2_PO_4_, 10 MgCl_2_, 0.5 CaCl_2_, 25 NaHCO_3_, 11 glucose, and myo-inositol. A whole-cell patch-clamp recording was performed in voltage-clamp mode on superficial layer II/III pyramidal neurons according to the similar method described previously (62). The external solution consisted of (in mM) 125 NaCl, 2.5 KCl, 1.25 NaH_2_PO_4_, 2 MgCl_2_, 1.6 CaCl_2_, 10 glucose, and 25 NaHCO_3_ saturated with 95% O_2_ and 5% CO_2_. Patch pipettes (8–10 MΩ) were filled with an internal solution of (in mM) 120 K-gluconate, 10 HEPES, 0.2 EGTA, 20 KCl, 2 MgCl_2_, 7 Na_2_-phosphocreatine, 4 Mg-ATP, and 0.3 Na_2_-GTP, pH adjusted to 7.3 with KOH (for mEPSC recordings) or 135 Cs methanesulfonate, 5 CsCl, 0.5 CaCl_2_, 2 MgCl_2_, 5 EGTA, 5 HEPES, and 4 Mg-ATP, pH adjusted to 7.3 with KOH (for mIPSC recordings). EPSCs and IPSCs were recorded at a holding potential of –70 mV in the presence of 1 μM tetrodotoxin (TTX) and 10 μM bicuculline methiodide (a GABA type A receptor antagonist) (for mEPSCs) and 0 mV in the presence of 1 μM TTX, 10 μM 2,3-dioxo-6-nitro-1,2,3,4-tetrahydrobenzo[f]quinoxaline-7-sulfonamide (a non-NMDA receptor antagonist), and 50 μM D-(-)-2-amino-5-phosphonopentanoic acid (an NMDA receptor antagonist) (for mIPSCs), respectively. Firing patterns were recorded in current-clamp mode. Current steps (0–120 pA, increasing in increments of 10 pA) were applied for 500 ms.

### Statistics.

Statistical significance was assessed using the JMP Pro 14 software program (SAS Institute Japan Ltd., Tokyo, Japan). Wilcoxon’s rank sum test was used as a nonparametric method, and Student’s *t* test, Welch’s *t* test, and a 1-way ANOVA were used for parametric data. Tests for normality were done by Shapiro-Wilk test. For contingency table statistics, Pearson’s χ^2^ test, Fisher’s exact test, or the Cochran-Armitage trend test were used. The Kruskal-Wallis test and Steel-Dwass test were sequentially used for multiple-comparison analyses of nonparametric data. Mouse survival ratios were analyzed by log rank tests. To compare the current injection-induced firing frequency between genotypes in patch-clamp tests, a 2-way repeated measures ANOVA was used. The statistical tests were 2 tailed. The threshold *P* values for statistical significance were < 0.05 (*), < 0.01 (**), < 0.001 (***), and < 0.0001 (****). Nonparametric data are shown as dot plots and box plots (center line, median; box limits, upper and lower quartiles; whiskers, 1.5 times interquartile range). Parametric data are shown as dot plots and bar graphs with error bars (standard deviation).

### Study approval.

All animal experimental procedures were reviewed and approved by Animal Care and Use Committee at Kyushu University (approval numbers A29-076-1, A28-014-1, A30-077-2, and A19-097-1).

## Author contributions

YK and DT wrote the manuscript and designed and carried out major experiments. YH and HN contributed to the patch-clamp analysis of neurons. NA and KS contributed to the quantification of inosine in RNA. YY contributed to the construction of the targeting vector. ST carried out the histopathological analyses. YN wrote the manuscript and designed the experiments.

## Supplementary Material

supplemental data

supplemental Video 1

supplemental Video 2

## Version 1 11/19/2020

Electronic publication
