# Supplementary material for: Neural stem cell–specific ITPA deficiency causes neural depolarization and epilepsy
Source: JCI Insight. 2020 Nov 19;5(22):e140229. doi: 10.1172/jci.insight.140229 (PMC7710303; doi:10.1172/jci.insight.140229)
Supplement: supplemental data [file jciinsight-5-140229-s007.pdf]

Supplemental data for Neural stem cell-specific ITPA deficiency causes neural depolarization and epilepsy by Koga et al.

1. Supplemental Figures.
2. Supplemental Tables.
3. Legends for Supplemental Movies.

Supplemental Figures

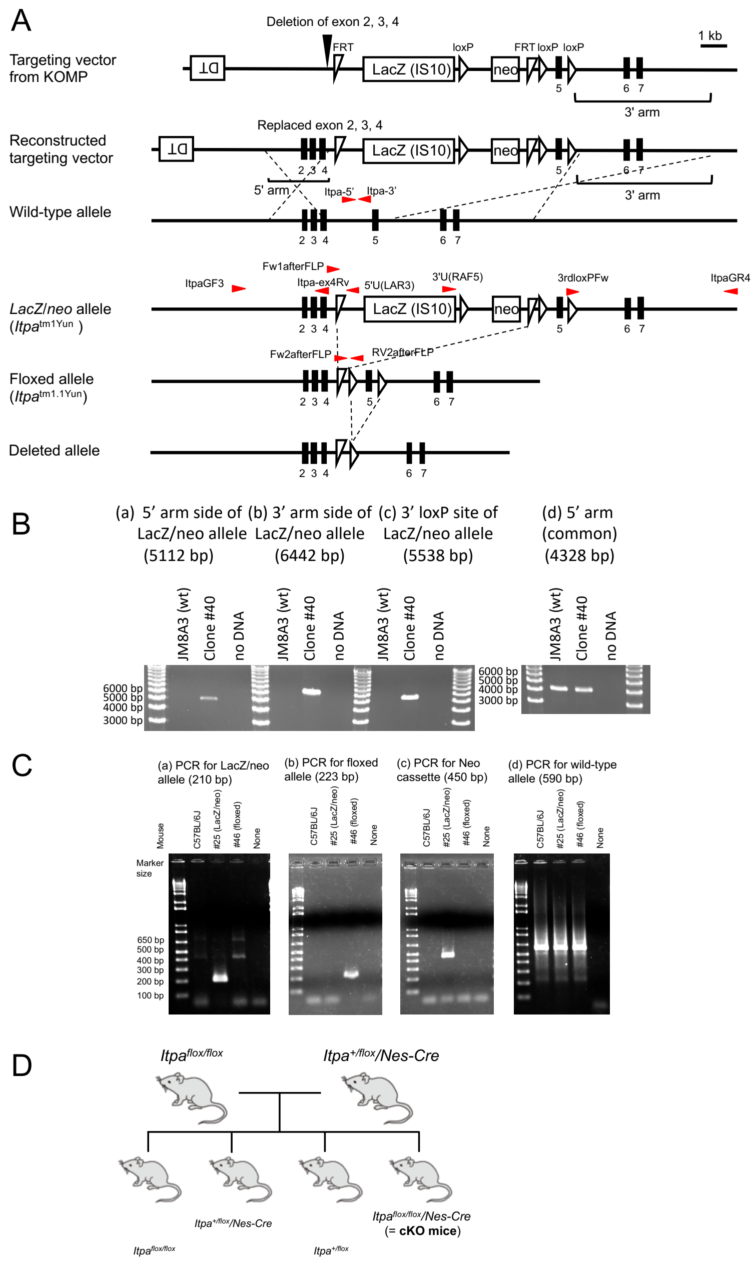

Supplemental Figure 1. Generation of *Itpa-flox* mice. (A) A schematic representation of the wild-type mouse *Itpa* allele, the targeting vectors before and after the reconstruction (targeting vector from KOMP and reconstructed targeting vector), the targeted *Itpa* allele with a LacZ/neo cassette (LacZ/neo allele or *Itpa*$^{\mathrm{tm1Yun}}$), the targeted *Itpa* allele without LacZ/neo cassette (floxed allele or *Itpa*$^{\mathrm{tm1.1Yun}}$), and the floxed *Itpa* allele after removal of exon 5 by Cre recombinase (deleted allele). Crossing and non-crossing dotted lines indicate the regions of homologous recombination and deletion, respectively. Exons, loxP sites, and FRT sites are indicated by numbered black boxes,

open isosceles triangles, and open right triangles, respectively. Oligo DNA primers for PCR genotyping are shown by red arrowheads. The position of unexpected deletion of exons 2, 3, 4 in the KOMP targeting vector is shown by a black arrowhead. (**B**) PCR for the LacZ/neo *Itpa* allele in ES cells. The LacZ/neo allele-specific PCR products for the 5' arm (5112 bp), 3' arm (6442 bp), and 3' loxP region (5538 bp), and common PCR product for the 5' arm region (4328 bp) of the LacZ/neo and wild alleles were prepared by genotyping PCR and separated by agarose gel electrophoresis. Genomic DNA extracted from wild-type JM8A3 ES cells and targeted ES clone #40 were used as templates. The PCR protocols are shown in the Methods section. (**C**) PCR for the LacZ/neo allele or the floxed allele in mice. PCR products specific for the LacZ/neo allele (a: 210 bp), the floxed allele (b: 223 bp), Neo cassette (c: 450 bp), or wild-type *Itpa* allele (d: 590 bp) were amplified by genotyping PCR and separated by agarose electrophoresis. Genomic DNA extracted from the tail of a C57BL/6J wild-type mouse, mouse #25 with a floxed *Itpa* allele with LacZ/neo, and mouse #46 with floxed *Itpa* allele without LacZ/neo were used as templates. The PCR protocols are shown in the Methods section. (**D**) Mating scheme to generate conditional knockout mice. To obtain neural stem cell-specific *Itpa* knockout mice (*Itpa*-cKO; *Itpa*$^{flox/flox/}$*Nes-Cre*), *Itpa*$^{flox/flox}$ mice were mated with *Itpa*$^{+/flox/}$*Nes-Cre* mice. As a result of this mating, we expected the birth of even numbers of mice across four different genotypes, including *Itpa*-cKO mice.

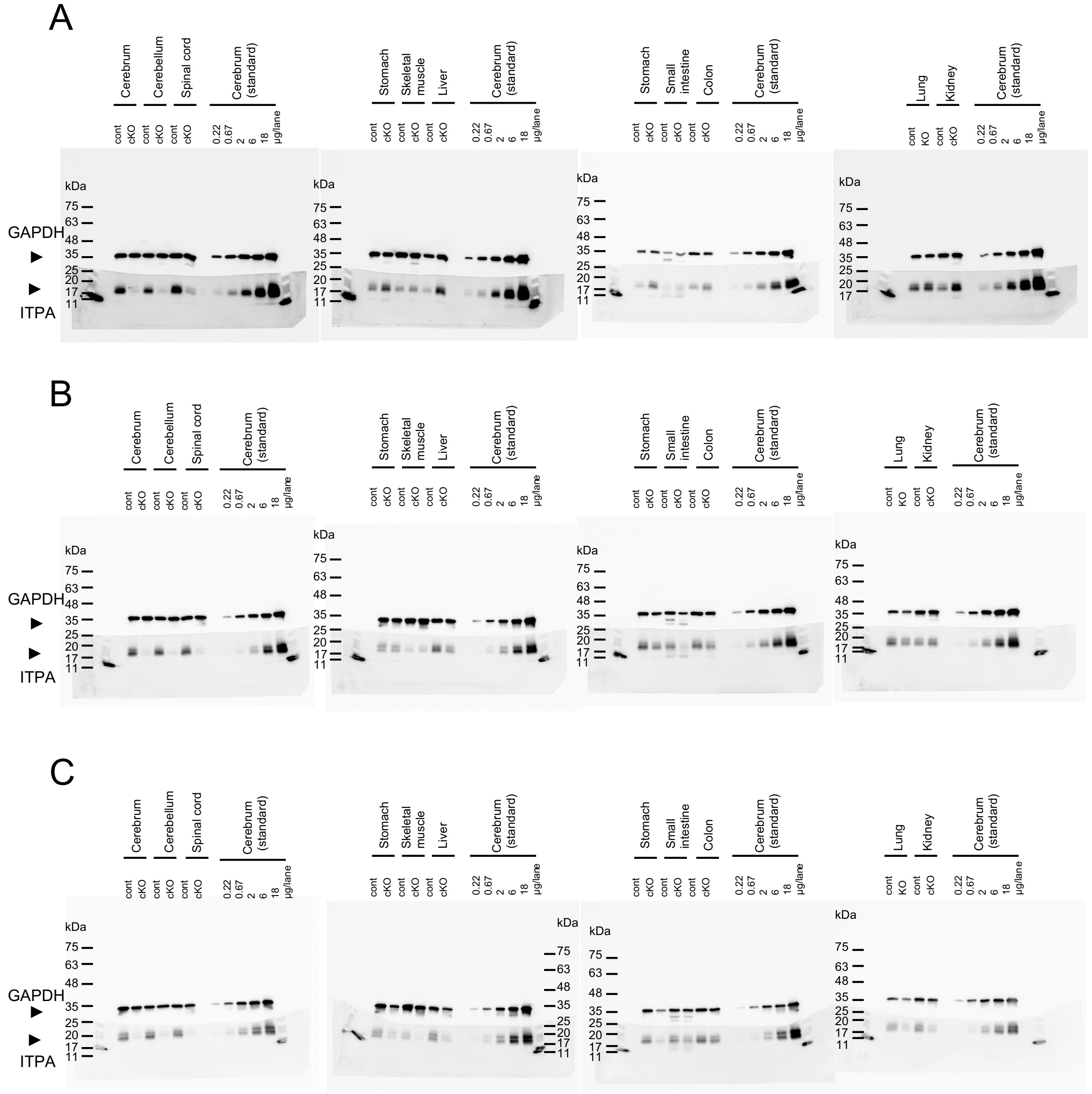

Supplemental Figure 2. Whole blot images of an immunoblot analysis of ITPA and GAPDH proteins. Whole blot images of an immunoblot analysis (Fig. 1B) of P16 or P17 male mouse tissues with anti-ITPA antiserum (lower parts) or anti-GAPDH antibody (upper parts). For each tissue, extracts from six mice (three control and three *Itpa*-cKO) were separated on three different blots (A, B, and C). On each blot, tissue extracts from one control mouse and one *Itpa*-cKO mouse were loaded with the common standard sample, as described in the "METHODS". The arrow heads show signals of ITPA and GAPDH proteins. Cont: control mouse (*Itpa*$^{flox/flox}$), cKO: conditional knockout mouse (*Itpa*$^{flox/flox}$/*Nes-Cre*).

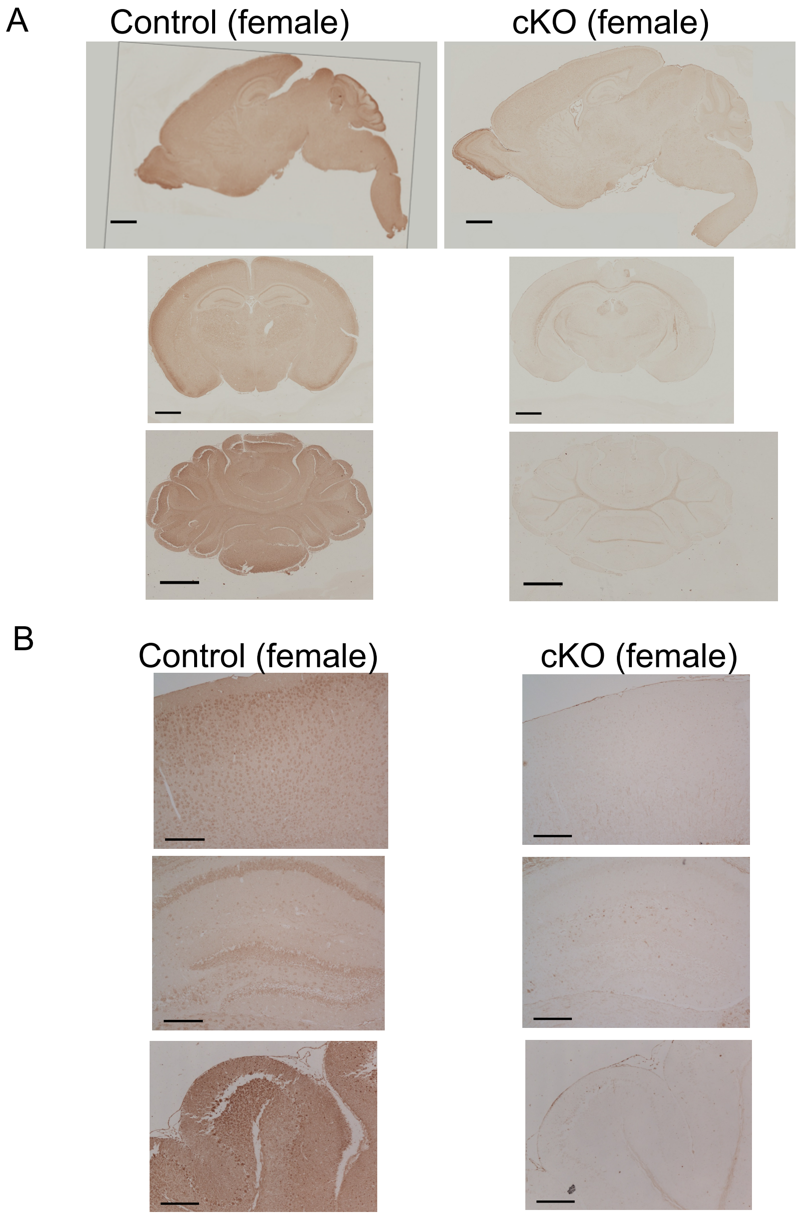

Supplemental Figure 3. Immunohistochemistry of P16 female mouse brains with anti-ITPA antibody. (**A**) Images of sagittal (upper), coronal including cerebrum (middle), and coronal including cerebellum (lower) sections of paraffin-embedded brains of control female mice (upper left; *Itpa*$^{+/flox}$/ *Nes-Cre*, middle and lower left; *Itpa*$^{flox/flox}$) and *Itpa*-cKO mice (right; *Itpa*$^{flox/flox}$/*Nes-Cre*,) are shown. Scale bar: 1 mm. (**B**) High-magnification images of the cerebral cortex (upper), dentate gyrus (middle), and cerebellar cortex (lower) from control (left; *Itpa*$^{flox/flox}$) and *Itpa*-cKO (right) are also shown. Scale bar: 200 µm.

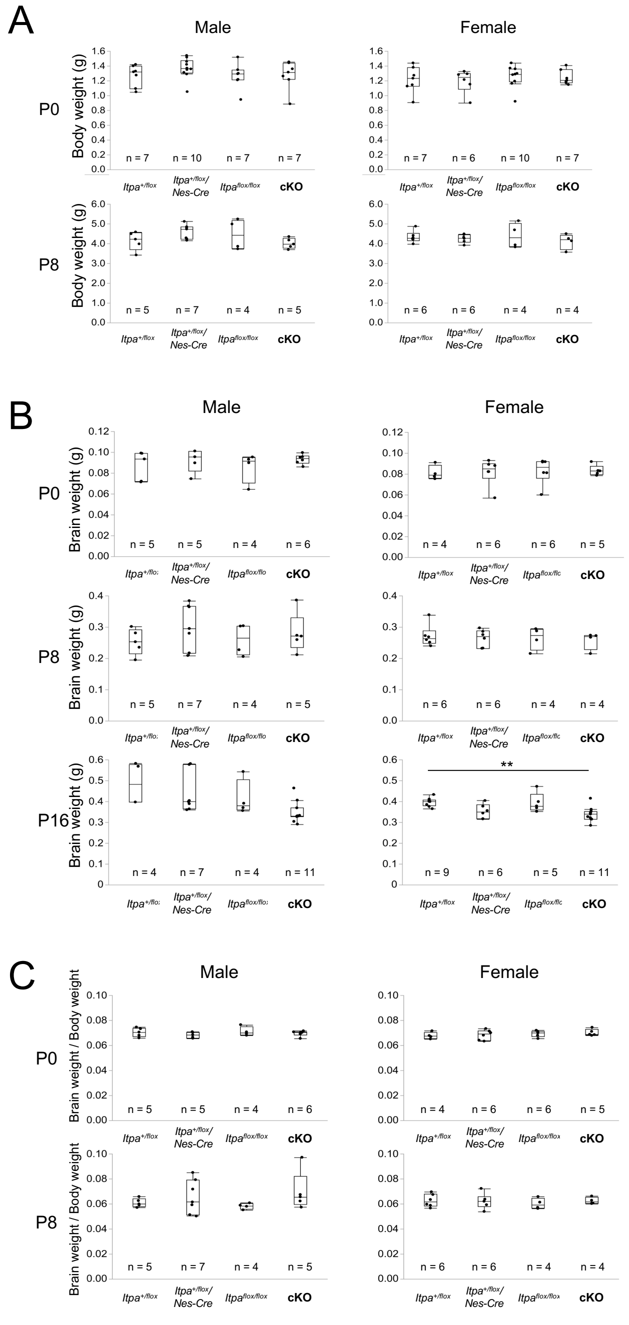

Supplemental Figure 4. Body and brain weights of control and *Itpa*-cKO mice on P0, P8 and P16. (A) The body weights of control and *Itpa*-cKO mice on P0 and P8 are shown as boxplots. Statistical analyses for P0 and P8 data were performed with the Kruskal-Wallis test: P0 male, P = 0.32; P0 female, P = 0.73; P8 male, P = 0.17; P8 female, P = 0.94. Data for P16 male and female mice are shown in Figure 2D. (**B**) The brain weights of control and *Itpa*-cKO mice on P0, P8, and P16 are shown as boxplots. Kruskal-Wallis test: P0 male, P = 0.65; P0 female, P = 0.59; P8 male, P = 0.70; P8 female, P = 0.97; P16 male, P = 0.0136; P16 female, P = 0.0031. Steel-Dwass test,

P16 female, Itpaflox/flox/Nes-Cre (Itpa-cKO) vs. Itpa+/flox ** *P* = 0.0099. (**C**) The Brain to body weight ratios of control and *Itpa*-cKO mice on P0 and P8 are shown as boxplots. Kruskal-Wallis test: P0 male, *P* = 0.74; P0 female, *P* = 0.64; P8 male, *P* = 0.35; P8 female, *P* = 0.81. Data for P16 male and female mice are shown in Figure 2E.

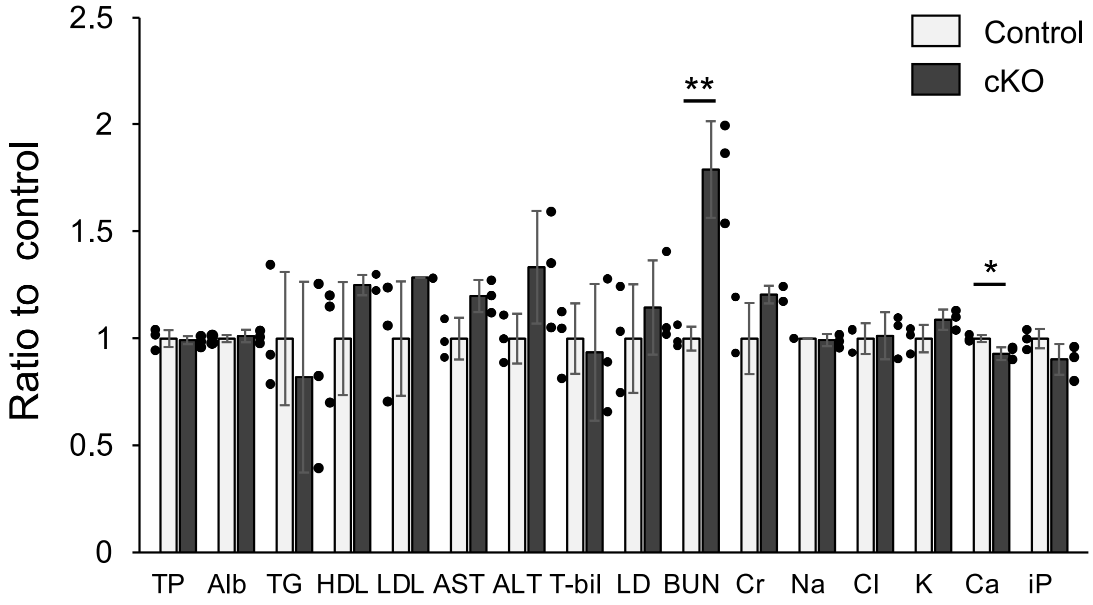

Supplemental Figure 5. Biochemical markers in peripheral blood serum. Relative levels of biochemical markers in the peripheral blood serum of P16 *Itpa*-cKO mice to P16 control (*Itpa*$^{flox/flox}$) mice (n =3) are shown as the mean ± SD. TP: total protein, Alb: albumin, TG: triglyceride, HDL: high-density lipoprotein cholesterol, LDL: low-density lipoprotein cholesterol, AST: aspartate transaminase, ALT: alanine transaminase, T-bil: total bilirubin, LD: lactate dehydrogenase, BUN: blood urea nitrogen, Cr: creatinine, Na: sodium ion, Cl: chloride ion, K: potassium ion, Ca: calcium ion, iP: inorganic phosphorus. Statistical analyses were performed with Student's *t*-test, BUN, ** $P = 0.0086$; Ca, * $P = 0.042$.

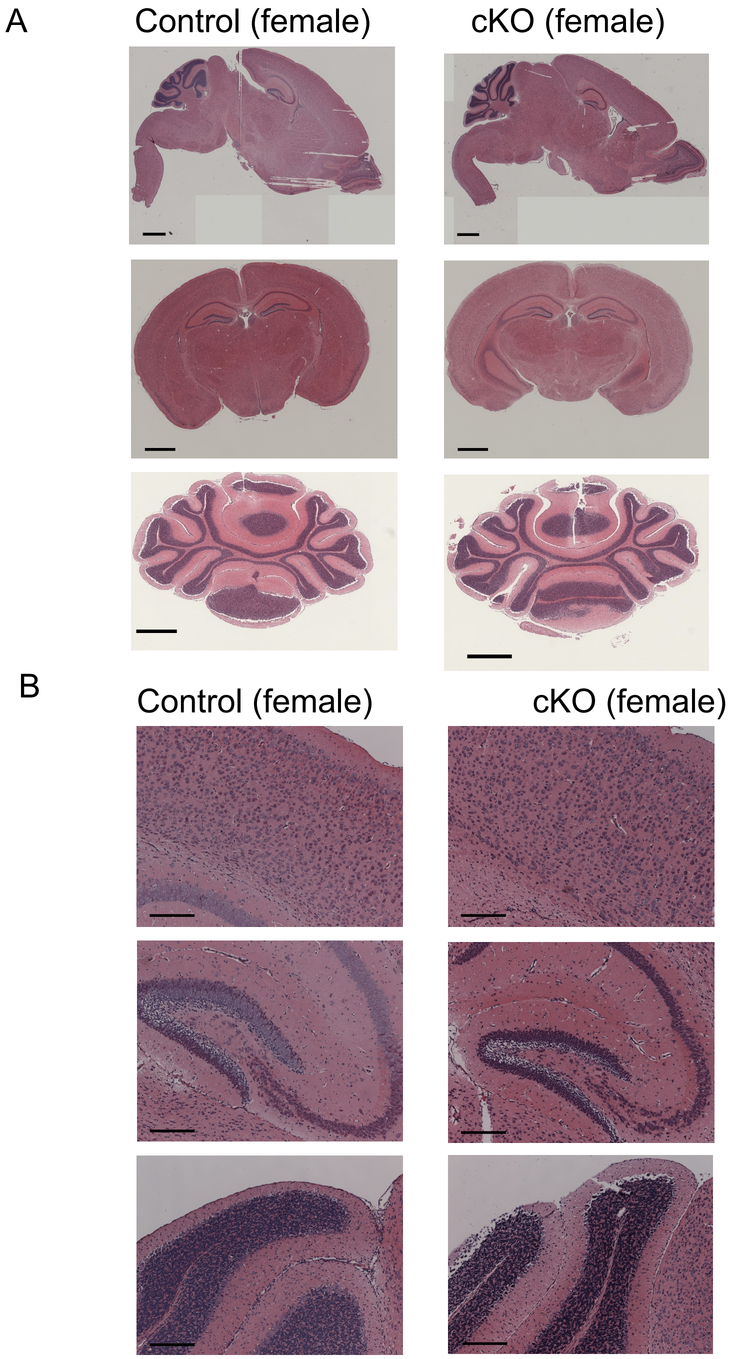

Supplemental Figure 6. Hematoxylin and eosin (H&E) staining of female mouse brain sections. (A). Sagittal thin sections (upper), coronal thin sections including cerebrum (middle) and coronal thin sections including cerebellum (lower) of paraffin-embedded whole brains from P16 control female mice (upper left; *Itpa*$^{+/flox/}$*Nes-Cre,* middle and lower left; *Itpa*$^{flox/flox}$) and *Itpa*-cKO female mice (right) were subjected to H&E staining. Scale bar: 1 mm. (**B**) High-magnification images of the above sagittal sections are shown for cerebral cortices (upper), dentate gyrus (middle), and cerebellar cortices (lower). Scale bar: 200 μm.

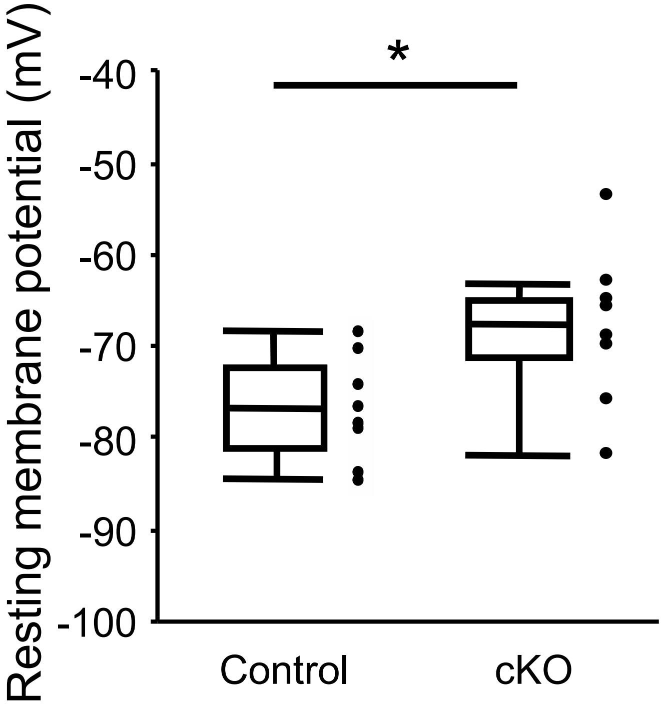

Supplemental Figure 7. Resting membrane potential of entorhinal cortex neurons of female mice. The resting membrane potential of entorhinal cortex neurons was analyzed by a whole-cell current-clamp test with mouse brain slices and is shown as box plots. Control cells (9 cells from one female P18 control mouse; *Itpa*$^{flox/flox}$) and *Itpa*-cKO cells (10 cells from one female P17 *Itpa*-cKO mouse) were analyzed. Statistical analysis was performed with Wilcoxon's rank-sum test; * P = 0.0178.

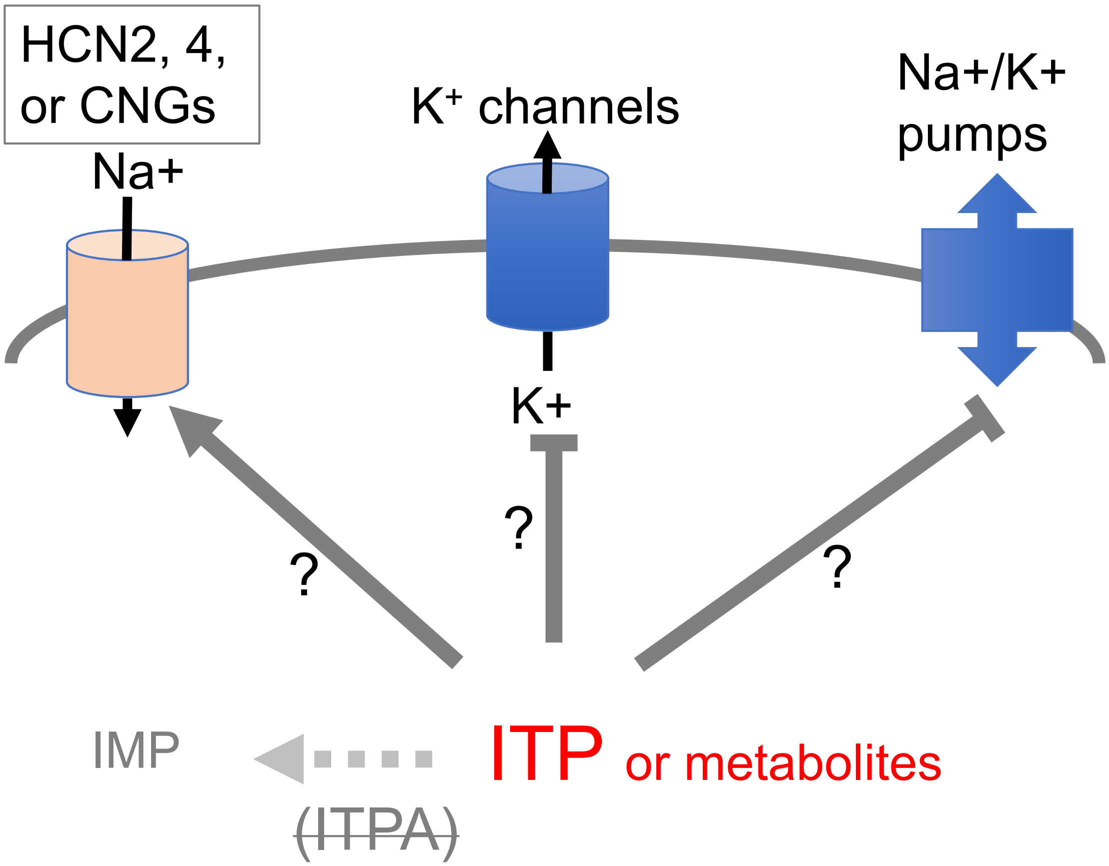

Supplemental Figure 8. Hypothesis of mechanism of depolarization. In ITPA-deficient neurons, accumulated ITP in cytosol may cause depolarization by the inhibition of Na+/K+ pumps or ion channels, such as leaky potassium ion channels, or by activation of ion channels, such as hyperpolarization-activated, cyclic nucleotide-gated ion channel (HCN) 2 or 4 or cyclic nucleotide gated channels (CNGs). These effects can be directly induced by ITP or an indirect reaction mediated by metabolites of ITP, such as RNA with inosine.

Supplemental Table 1. Oligo DNAs

Oligo DNA name	Sequence	Purpose
NF3	CATTCTGGAAGCTGAAATAGGAGGAC	Sequencing of deletion region
NR5	GCTTTACTCAGGCCAGGATGTCATATC	Sequencing of deletion region
NFGItpaCKO1	TGTGCCGAAATGGTCCATCA	Sequencing of IS10 insertion
NFGItpaCKO2	TAAGTGAAGCGACCCGCATT	Sequencing of IS10 insertion
NFGItpaCKO4	AAAGGCAACTTTCGGAACGG	Sequencing of IS10 insertion
NFGItpaCKO5	GATGGATTGCACGCAGGTTC	Sequencing of IS10 insertion
NFGItpaCKO6	GACCGCTTCCTCGTGCTTTA	Sequencing of IS10 insertion
NFGItpaCKO7	TCATGCTGGAGTTCTTCGCC	Sequencing of IS10 insertion
IS10Fw1	GGAAGAAGGCACATGGCTGA	Sequencing of IS10 insertion
IS10Fw2	CATATGGGGATTGGTGGCGA	Sequencing of IS10 insertion
IS10Rv1	CCCCCTGCCCGGTTATTATT	Sequencing of IS10 insertion
ItpaEx234Fw3	GCGATCGCAAAGACCCAGAGGCTTTA	Exon 2,3,4 PCR
ItpaEx234Rv3	CTTAAGGGAGGTACTAGGTCCCTTGC	Exon 2,3,4 PCR
Itpa-GF3	CTACCTACTTCCGCCTCCGGCCGGAAGTAC	ES the LacZ/neo 5'-arm PCR and ES common PCR
5'Universal(LAR3)	CACAACGGGTTCTTCTGTTAGTCC	ES the LacZ/neo 5'-arm PCR and Mouse LacZ/neo allele PCR
3'Universal(RAF5)	CACACCTCCCCCTGAACCTGAAAC	ES the LacZ/neo 3'-arm PCR
Itpa-GR4	GCTACATGTGGCTAAGTTAATGACAAC	ES the LacZ/neo 3'-arm PCR and ES 3'-loxP PCR
3rdloxPFw	GATGGCGCAACGCAATTAATGA	ES 3'-loxP PCR
Itpa-ex4Rv	GTCCCCCAAGTGCGTTAAAGCACAGACAGG	ES common PCR
Fw1afterFLP	TCCCTTAAGGCGCATAACGA	Mouse LacZ/neo allele PCR
Fw2afterFLP	GGCGCATAACGATACCACGAT	Mouse floxed allele PCR
Rv2afterFLP	AACATCTCCGCCTACTGCG	Mouse floxed allele PCR
NER1	GCTCTTCGTCCAGATCATCC	NEO cassette PCR
NEL2	GATTGCACGGAGGTTCTCCG	NEO cassette PCR
FLP	CACCACCTAAGGTCCTGGTTC	Mouse CAG-FLPe PCR
Flp-IntR	CTGCTTCTTCCGATGATTCG	Mouse CAG-FLPe PCR
IMR0015	CAAATGTTGCTTGTCTGGTG	Mouse CAG-FLPe PCR
IMR0016	GTCAGTCGAGTGCACAGTTT	Mouse CAG-FLPe PCR
Cre-1	ACA TGT TCA GGG ATC GCC AG	Mouse Cre PCR
Cre-2	TAA CCA GTG AAA CAG CAT TGC	Mouse Cre PCR
Itpa-5'	TTCAGAGATGATATGACATCCTGG	Mouse wt allele PCR
Itpa-3'	TGCTCTGCCCTACACCCAACAGGG	Mouse wt allele PCR
mITPA_C Nco_beta4_Fw#2	CCGCCATGGAAGACAAATCGGCCTATGC	Mouse ITPA-C cDNA PCR
mITPA_C_Hind_Rv	CGCAAGCTTGCATGCCTGCAGGTCGACTCTAG	Mouse ITPA-C cDNA PCR

Supplemental Table 2. Detail of audiogenic seizure tests.

Genotype	Sex	Induced seizure	
		(+)	(-)
Itpa$^{+/flox}$	male	0	2
Itpa$^{+/flox}$*/Nes-Cre*	male	0	5
Itpa$^{flox/flox}$	male	0	5
cKO	male	5	0
Itpa$^{+/flox}$	female	0	4
Itpa$^{+/flox}$*/Nes-Cre*	female	0	7
Itpa$^{flox/flox}$	female	0	2
cKO	female	7	0

Legends for Supplemental Movies

Supplemental Movie 1. Spontaneous seizure in an *Itpa* conditional knockout mouse. A P14 female mouse showed spontaneous seizure before weaning. The mouse was soon transferred to a new empty transparent plastic container, and her behavior was recorded.

Supplemental Movie 2. Audiogenic seizure in an *Itpa* conditional knockout mouse. A P16 male mouse showed seizure when exposed to audio stress.
